# Crystal structure of a host–guest complex of the tris-urea receptor, 3-(4-nitro­phen­yl)-1,1-bis­{2-[3-(4-nitro­phen­yl)ureido]eth­yl}urea, that encapsulates hydrogen-bonded chains of di­hydrogen phosphate anions with separate tetra-*n*-butyl­ammonium counter-ions

**DOI:** 10.1107/S2056989019001336

**Published:** 2019-02-05

**Authors:** Ruyu Wang, Xi Shu, Yu Fan, Shoujian Li, Yongdong Jin, Chuanqin Xia, Chao Huang

**Affiliations:** a College of Chemistry, Sichuan University, Chengdu 610064, People’s Republic of China

**Keywords:** crystal structure, tris-urea, tetra-*n*-butyl­ammonium, di­hydrogen phosphate, anionic polymer, hydrogen bonding, supra­molecular three-dimensional structure

## Abstract

The title compound, C_25_H_25_N_9_O_9_·C_16_H_36_N^+^·H_2_PO_4_^−^ (I) or (C_25_H_25_N_9_O_9_)·(*n*-Bu_4_N^+^)·(H_2_PO_4_^−^) (systematic name: 3-(4-nitro­phen­yl)-1,1-bis­{2-[3-(4-nitro­phen­yl)ureido]eth­yl}urea tetra­butyl­ammonium di­hydrogen phosphate), comprises a tris-urea receptor (**R**), a di­hydrogen phosphate anion and a tetra-*n*-butyl­ammonium cation. It crystallizes with two independent formula units in the asymmetric unit. The conformations of the two tris-urea receptors are stabilized by N—H⋯O and C—H⋯O intra­molecular hydrogen bonds. Each di­hydrogen phosphate anion has two O—H⋯O inter­molecular hydrogen-bonding inter­actions with the other di­hydrogen phosphate anion. Inversion-related di-anion units are linked by further O—H⋯O hydrogen bonds, forming a chain propagating along the *a*-axis direction. Each di­hydrogen phosphate anion makes a total of four N—H⋯O(H_2_PO_4_^−^) hydrogen bonds with two ureido subunits from two different tris-urea receptors, hence each tris-urea receptor provides the two ureido subunits for the encapsulation of the H_2_PO_4_^−^ hydrogen-bonded chain. There are numerous inter­molecular C—H⋯O hydrogen bonds present involving both receptor mol­ecules and the tetra-*n*-butyl­ammonium cations, so forming a supra­molecular three-dimensional structure. One of the butyl groups and one of the nitro groups are disordered over two positions of equal occupancy.

## Abstract 2

The title compound is a host–guest complex of the tris-urea receptor, 3-(4-nitro­phen­yl)-1,1-bis­{2-[3-(4-nitro­phen­yl)ureido]eth­yl}urea, encapsulating a hydrogen-bonded chain of di­hydrogen phosphate anions.

## Chemical context   

Anions play an important role in many chemical, catalysis, environmental and biological systems (Sessler *et al.*, 2006 ▸; Vickers & Beer, 2007 ▸; Beer & Gale, 2001 ▸). The use of urea-based receptors as hydrogen-bond donors in anion recognition has attracted much attention (Xu *et al.*, 2017 ▸; Amendola *et al.*, 2006 ▸; Hoque & Das, 2017 ▸; Bregović *et al.*, 2015 ▸; Li *et al.*, 2010 ▸). In particular, considerable research efforts have been devoted to the designation of receptors containing a ureido subunit (which selectively recognizes fluoride ions), such as 1,3-bis­(4-nitro­phen­yl) urea (Boiocchi *et al.*, 2004 ▸), or a urea subunit equipped with two naphthalenimide moieties (Esteban-Gómez *et al.*, 2005 ▸), and thio­urea or urea-based indole conjugated ligands (Bose & Ghosh, 2010 ▸). Recently, tris­(2-amino­eth­yl)amine (tren)-based tripodal urea or thio­urea receptors for the recognition and separation of anions have been investigated (Arunachalam & Ghosh, 2011 ▸; Custelcean, 2013 ▸; Dey *et al.*, 2016 ▸; Hay *et al.*, 2005 ▸). However, tris-urea receptors have been rarely studied to date. In our ongoing research on nitro­gen-rich organic ligands (Wang *et al.*, 2015 ▸) and the design and synthesis of ureido receptors (Huang *et al.*, 2017 ▸), we report herein the synthesis of the title tris-urea receptor, 3-(4-nitro­phen­yl)-1,1-bis­{2-[3-(4-nitro­phen­yl)ureido]eth­yl}urea (**R**), based on *p*-nitro­phenyl substituents, and the crystal structure of its complex with tetra-*n*-butyl­ammonium di­hydrogen phosphate. Inter­estingly, a one-dimensional hydrogen-bonded polymeric structure is formed *via* hydrogen bonds between di­hydrogen phosphate anions, and this anionic polymer is surrounded by and linked to the tris-urea receptors through ureido N—H⋯O hydrogen bonds.
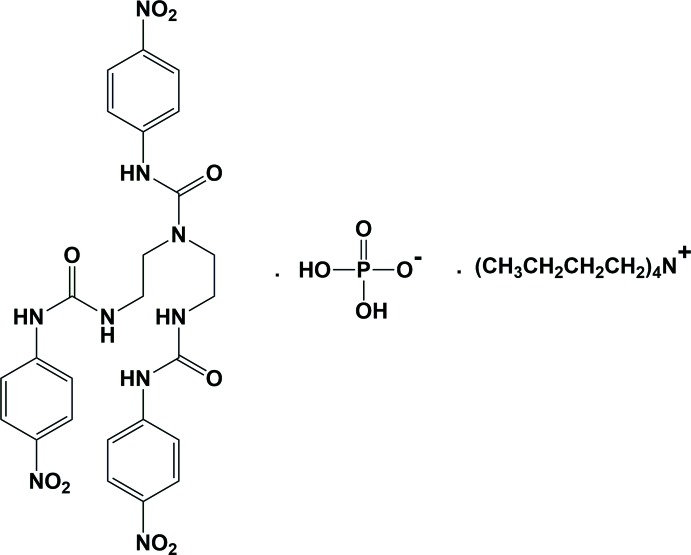


## Structural commentary   

**Figure 1:**
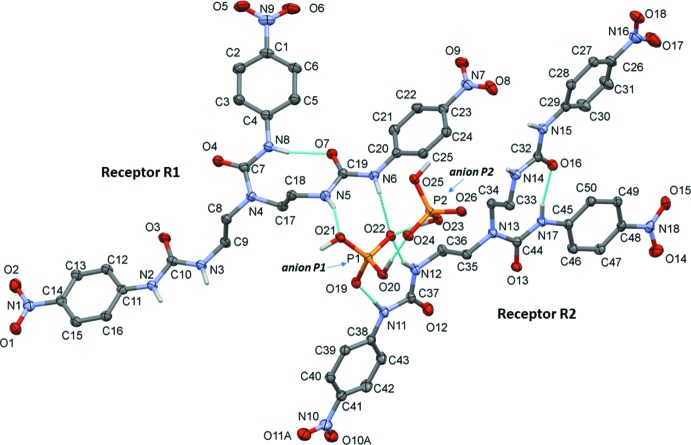
The mol­ecular structure of the title complex, with atom labellling. Displacement ellipsoids are drawn at the 30% probability level. For clarity, the tetra-*n*-butyl­ammonium cations, the disordered NO_2_ O atoms and the C-bound hydrogen atoms have been omitted.

The title compound crystallizes with two independent formula units in the asymmetric unit. The mol­ecular structure of the two tris-urea receptors (*R*1 and *R*2) and the di­hydrogen phosphate anions (*P*1 and *P*2) are illustrated in Fig. 1 ▸. In each receptor an intra­molecular N—H⋯O hydrogen bond is present (N8—H8*N*⋯O7 in *R*1 and N17—H17*N*⋯O16 in *R*2), each forming an *S*(9) ring motif,. Four intra­molecular C—H⋯O hydrogen bonds are also present in receptor *R*1 and three in *R*2 (Fig. 1 ▸ and Table 1 ▸). Both receptors display a *cis* orientation of the urea subunits (N2/N3 and N5/N6 in *R*1, and N11/N12 and N14/N15 in *R*2). The urea subunits N5/N6 in *R*1 and N11/N12 in *R*2 are orientated towards the di­hydrogen phosphate ions (*P*1 and *P*2) forming a 2:2 adduct *via* N—H⋯O hydrogen bonds with anion *P*1 and enclosing 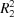
(8) ring motifs (Table 1 ▸ and Fig. 1 ▸). Both NH functions of each urea subunit are *trans* to the C=O group across the respective C—N bond. Anions *P*1 and *P*2 inter­act with each other *via* two O—H⋯O hydrogen bonds (O20—H20*O*⋯O24 and O23—H23*O*⋯O22), enclosing an 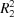
(8) ring motif (Fig. 1 ▸ and Table 1 ▸). The dihedral angle between the urea plane [N—C(=O)—N] and the benzene ring to which it is attached vary from 6.66 (14) to 18.96 (14)° in *R*1 and from 6.87 (14) to 13.82 (14)° in *R*2. The dihedral angle between the nitro group and the benzene ring to which it is attached also vary, from 7.1 (3) to 13.4 (4)° in *R*1 and from 8.3 (4) to 16.7 (7)° in *R*2.

## Supra­molecular features   

**Figure 2:**
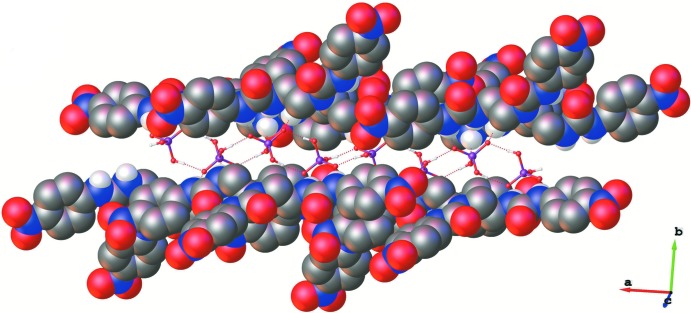
A partial view along the *b* axis of the crystal packing of the title complex. The receptors *R*1 and *R*2 are drawn in space-filling mode. The H atoms not involved in the O—H⋯O hydrogen bonds have been omitted.

**Figure 3:**
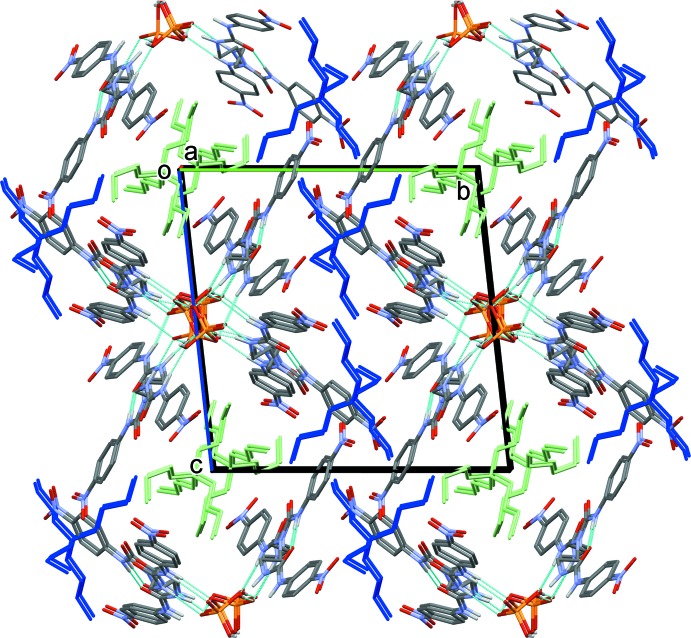
Crystal packing of the title complex, viewed along the *a* axis. The tetra-*n*-butyl­ammonium cations are shown in blue (involving atom N19) and green (involving atom N20). The H atoms not involved in the O—H⋯O hydrogen bonds have been omitted.

In the crystal, the two H_2_PO_4_^−^ anions (*P*1 and *P*2), that are linked by O—H⋯O hydrogen bonds O20—H20*O*⋯O24 and O23—H23*O*⋯O22, are further linked to inversion-related anions *via* hydrogen bonds O21—H21*O*⋯O19^i^ and O25—H25*O*⋯O26^ii^ (Table 1 ▸). This results in the formation of a polymer chain propagating along the *a-*axis direction (Fig. 2 ▸). The receptor mol­ecules are linked to this chain *via* hydrogen bonds N5—H5*N*⋯O21, N6—H6*N*⋯O22, N11—H11*N*⋯O19, and N12—H12*N*⋯O22 (Fig. 1 ▸), and by hydrogen bonds N2—H2*N*⋯O26^i^, N3—H3*N*⋯O24^i^, N14—H14*N*⋯O25^ii^ and N15—H15*N*⋯O24^ii^ (Table 1 ▸). Finally, there are numerous inter­molecular C—H⋯O hydrogen bonds present involving both receptor mol­ecules and the tetra-*n*-butyl­ammonium cations, so forming a supra­molecular three-dimensional structure (Fig. 3 ▸ and Table 1 ▸).

## Database survey   

The crystal structure of the receptor **R**, with or without *para*-substitution of a nitro group, has not previously been reported. A search of the Cambridge Structural Database (Version 5.40, November 2018; Groom *et al.*, 2016 ▸) for tetra-*n*-butyl­ammonium di­hydrogen phosphate yielded four hits, essentially concerning the tris-urea receptor based on tris­(2-amino­eth­yl)amine (tren). One of these compounds, tetra-*n*-butyl­ammonium tris­(2-(*N*-perfluoro­phenyl­ureaylato)eth­yl)amine di­hydrogen phosphate di­methyl­formamide monosolvate, encapsulates a dimer of H_2_PO_4_^−^ anions forming a pseudo-dimeric cage *via* sixteen hydrogen bonds and two weak anion⋯π inter­actions (CSD refcode CITYOU; Lakshminarayanan *et al.*, 2007 ▸). Another example is, *N*,*N′*,*N*′′-[nitrilo­tris­(ethane-2,1-di­yl)]tris­(*N*′-phenyl­urea) tetra-*n*-butyl­ammonium di­hydrogen phosphate (YICHUQ; Manna & Das, 2018 ▸). Here too, a dimer of H_2_PO_4_^−^ anions is encapsulated by the receptor.

Chiral anion receptors with two enanti­omeric forms *R*,*R* and *S*,*S* based on a 1,2-cyclo­hexane moiety appended by two *p*-nitro­phenyl­urea subunits have been reported. One such compound is bis­(tetra-*n*-butyl­ammonium) bis­(di­hydrogen phosphate) (*R*,*R*)-1-(4-nitro­phen­yl)-3-{2-[3-(4-nitro­phen­yl)ureido]cyclo­hex­yl}urea aceto­nitrile monosolvate (DASNUH; Amendola *et al.*, 2005 ▸). The nature of the *R*,*R*-enanti­omer⋯H_2_PO_4_^−^ inter­actions is characterized by infinite di­hydrogen phosphate chains along the *a-*axis direction, as observed in the crystal structure of the title complex. Specif­ically, each di­hydrogen phosphate ion inter­acts with two adjacent H_2_PO_4_^−^ ions and with only one *R*,*R*-enanti­omer *via* two hydrogen bonds. The hydrogen bonds in this enanti­omeric complex are similar to those in the title complex, with the H⋯acceptor distance between the urea N—H group and the di­hydrogen phosphate oxygen atom varying between *ca* 1.98 and 2.19 Å in DASNUH compared to a range of 1.96 (2)–2.21 (2) Å in the title compound. The O—H⋯O hydrogen bonds involving the H_2_PO_4_^−^ anions are also very similar: 1.62 (3) and 1.63 (4) Å in DASNUH, while they vary from 1.59 (2) to 1.66 (2) Å in the title complex.

## Synthesis and crystallization   

**Synthesis of 3-(4-nitro­phen­yl)-1,1-bis­{2-[3-(4-nitro­phen­yl)ureido]eth­yl}urea (R):** In a 250 ml round-bottom flask, di­ethyl­enetri­amine (0.32 ml, 2.97 mmol) dissolved in 100 ml of dry CH_2_Cl_2_ was added dropwise under vigorous stirring to a solution of 20 ml of dry CH_2_Cl_2_ containing *p*-nitro­benzene iso­cyanate (1.64 g, 9.98 mmol). Subsequently, the reaction mixture was allowed to reflux for 24 h. A yellowish solid was collected by filtration and washed using sequentially CH_2_Cl_2_ (3 × 70 ml), a solvent mixture (CH_2_Cl_2_/THF = 4:1, 3 × 70 ml) and diethyl ether (3 × 70 ml). The solid was then dried *in vacuo* overnight to afford the receptor **R** as a light-brown powder (yield: 1.52 g; 85.6%; m.p. 512.3–513.8 K). FT–IR (KBr, cm^−1^): 3339, 1679, 1606, 1559, 1501, 1330. ^1^H NMR (400 MHz, DMSO-*d*_6_) in ppm: δ = 9.42 (*s*, 2H), 9.23 (*s*, 1H), 8.10 (*t*, *J* = 9.9 Hz, 6H), 7.76 (*d*, *J* = 8.9 Hz, 2H), 7.59 (*d*, *J* = 8.9 Hz, 4H), 6.60 (*t*, *J* = 5.8 Hz, 2H), 3.51 (*t*, *J* = 6.5 Hz, 4H). ^13^C NMR (100 MHz, DMSO-*d*_6_) in ppm: δ = 154.99, 154.67, 147.34, 146.94, 140.72, 140.48, 125.09, 124.70, 118.13, 116.95, 46.63, 40.15–38.89, 38.19. HRMS (ESI^+^): calculated for C_25_H_25_N_9_O_9_Na [*M* + Na]^+^ 618.1673 found 618.1678.

**Synthesis of the title complex (I):** Tetra-*n*-butyl­ammonium di­hydrogen phosphate (1.68 mmol) was added to 5 ml of a DMF solution of **R** (0.168 mmol) and the mixture was stirred for 2 h. After filtration the solution was left to evaporate slowly and yielded colourless prismatic crystals of the title complex within three weeks.

## Refinement   

Crystal data, data collection and structure refinement details are summarized in Table 2 ▸. H atoms bonded to O and N were located in a difference-Fourier map and refined with distance restraints: O—H = 0.93 and N—H = 0.90 Å, with *U*_iso_(H) = 1.5*U*_eq_(O) and 1.2*U*_eq_(N). The C-bound H atoms were positioned geometrically and refined using a riding model: C—H = 0.95–0.99 Å, with *U*_iso_(H) = 1.5*U*_eq_(C-meth­yl) and 1.2*U*_eq_(C) for other H atoms.

One of the butyl moieties (C79—C80—C81—C82) is disordered over two positions with equal occupancies. The C—C distances were refined with the restraint of 1.515 (4) Å. The displacement parameters of the C79*A*/C79*B*, C80*A*/C80*B*, C81*A*/C81*B*, and C82*A*/C82*B* atoms of the disordered fragment were restrained to be similar (Sheldrick, 2015*b* ▸). Also, one of the nitro groups (O11—N10—O10) was disordered over two positions with equal occupancies. The N—O distances were refined with the restraint of 1.230 (4) Å. The displacement parameters of the O10*A*/O11*A* and O10*B*/O11*B* atoms of the disordered fragment were restrained to be similar (Sheldrick, 2015*b* ▸).

## Supplementary Material

Crystal structure: contains datablock(s) I, Global. DOI: 10.1107/S2056989019001336/kq2020sup1.cif

Structure factors: contains datablock(s) I. DOI: 10.1107/S2056989019001336/kq2020Isup3.hkl

Supporting information file. DOI: 10.1107/S2056989019001336/kq2020Isup3.cml

CCDC reference: 1893140

Additional supporting information:  crystallographic information; 3D view; checkCIF report

## Figures and Tables

**Table 1:** Hydrogen-bond geometry (Å, °)

*D*—H⋯*A*	*D*—H	H⋯*A*	*D*⋯*A*	*D*—H⋯*A*
N8—H8*N*⋯O7	0.90 (3)	2.07 (3)	2.955 (3)	168 (3)
N17—H17*N*⋯O16	0.90 (2)	2.21 (2)	3.100 (3)	170 (2)
N5—H5*N*⋯O21	0.90 (2)	2.04 (2)	2.915 (3)	164 (3)
N6—H6*N*⋯O22	0.90 (2)	2.10 (2)	2.986 (3)	168 (2)
N11—H11*N*⋯O19	0.90 (2)	1.96 (2)	2.854 (3)	175 (2)
N12—H12*N*⋯O22	0.90 (2)	2.21 (2)	3.058 (3)	157 (3)
O20—H20*O*⋯O24	0.93 (2)	1.66 (2)	2.583 (2)	178 (3)
O23—H23*O*⋯O22	0.93 (2)	1.64 (2)	2.568 (2)	176 (2)
O21—H21*O*⋯O19^i^	0.93 (2)	1.61 (2)	2.528 (2)	173 (3)
O25—H25*O*⋯O26^ii^	0.93 (2)	1.59 (2)	2.501 (2)	166 (3)
N2—H2*N*⋯O26^i^	0.90 (2)	2.08 (2)	2.977 (3)	174 (2)
N3—H3*N*⋯O24^i^	0.90 (2)	2.09 (2)	2.931 (3)	155 (3)
N14—H14*N*⋯O25^ii^	0.90 (2)	2.03 (2)	2.930 (3)	175 (2)
N15—H15*N*⋯O24^ii^	0.90 (2)	2.10 (2)	2.986 (3)	168 (2)
C3—H3⋯O4	0.95	2.17	2.789 (4)	121
C5—H5⋯O7	0.95	2.53	3.279 (4)	136
C12—H12⋯O3	0.95	2.23	2.844 (3)	122
C21—H21⋯O7	0.95	2.31	2.872 (4)	118
C30—H30⋯O16	0.95	2.32	2.903 (4)	119
C43—H43⋯O12	0.95	2.32	2.910 (4)	120
C46—H46⋯O13	0.95	2.19	2.810 (3)	122
C18—H18*A*⋯O1^iii^	0.99	2.42	3.345 (4)	156
C30—H30⋯O6^iv^	0.95	2.58	3.265 (4)	129
C33—H33*B*⋯O11*A* ^iii^	0.99	2.58	3.235 (12)	123
C39—H39⋯O21^i^	0.95	2.52	3.446 (3)	164
C54—H54*A*⋯O15^v^	0.99	2.46	3.447 (4)	173
C63—H63*A*⋯O3	0.99	2.42	3.379 (4)	164
C63—H63*B*⋯O4	0.99	2.26	3.003 (4)	131
C64—H64*B*⋯O15^v^	0.99	2.46	3.348 (4)	149
C70—H70*B*⋯O10*A* ^iii^	0.98	2.47	3.289 (6)	141
C75—H75*A*⋯O13	0.99	2.31	3.158 (4)	143
C75—H75*B*⋯O1^vi^	0.99	2.58	3.514 (4)	157

Symmetry codes: (i) 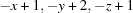; (ii) 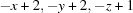; (iii) 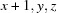; (iv) 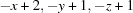; (v) 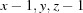; (vi) 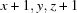.

**Table 2:** Experimental details

Crystal data	
Chemical formula	C_25_H_25_N_9_O_9_·C_16_H_36_N^+^·H_2_PO_4_^−^
*M*_r_	934.98
Crystal system, space group	Triclinic, *P* 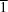
Temperature (K)	179
*a*, *b*, *c* (Å)	15.2043 (3), 17.4555 (5), 18.1232 (4)
α, β, γ (°)	83.797 (2), 79.818 (2), 89.191 (2)
*V* (Å^3^)	4706.3 (2)
*Z*	4
Radiation type	Cu *K*α
μ (mm^−1^)	1.13
Crystal size (mm)	0.40 × 0.40 × 0.10

Data collection	
Diffractometer	Agilent New Gemini, Dual, Cu at zero, EosS2
Absorption correction	Multi-scan (*CrysAlis PRO*; Agilent, 2014 ▸)
*T*_min_, *T*_max_	0.660, 0.880
No. of measured, independent and observed [*I* > 2σ(*I*)] reflections	50539, 18359, 15323
*R*_int_	0.040
(sin θ/λ)_max_ (Å^−1^)	0.619

Refinement	
*R*[*F* ^2^ > 2σ(*F* ^2^)], *wR*(*F* ^2^), *S*	0.065, 0.160, 1.01
No. of reflections	18359
No. of parameters	1237
No. of restraints	26
H-atom treatment	H atoms treated by a mixture of independent and constrained refinement
Δρ_max_, Δρ_min_ (e Å^−3^)	0.96, −0.85

Computer programs: *CrysAlis PRO* (Agilent, 2014 ▸), *SHELXT* (Sheldrick, 2015*a* ▸), *SHELXL* (Sheldrick, 2015*b* ▸), *OLEX2* (Dolomanov *et al.*, 2009 ▸), *Mercury* (Macrae *et al.*, 2008 ▸), *PLATON* (Spek, 2009 ▸) and *publCIF* (Westrip, 2010 ▸).

## supplementary crystallographic information

### Crystal data

**Table:**

C_25_H_25_N_9_O_9_·C_16_H_36_N^+^·H_2_PO_4_^−^	*Z* = 4
*M**_r_* = 934.98	*F*(000) = 1992
Triclinic, *P*1	*D*_x_ = 1.320 Mg m^−^^3^
*a* = 15.2043 (3) Å	Cu *K*α radiation, λ = 1.54184 Å
*b* = 17.4555 (5) Å	Cell parameters from 22142 reflections
*c* = 18.1232 (4) Å	θ = 4.2–72.6°
α = 83.797 (2)°	µ = 1.13 mm^−^^1^
β = 79.818 (2)°	*T* = 179 K
γ = 89.191 (2)°	Prism, colourless
*V* = 4706.3 (2) Å^3^	0.40 × 0.40 × 0.10 mm

### Data collection

**Table:**

Agilent New Gemini, Dual, Cu at zero, EosS2 diffractometer	18359 independent reflections
Radiation source: Enhance (Cu) X-ray Source	15323 reflections with *I* > 2σ(*I*)
Detector resolution: 15.9595 pixels mm^-1^	*R*_int_ = 0.040
ω scans	θ_max_ = 72.7°, θ_min_ = 4.5°
Absorption correction: multi-scan (*CrysAlisPro*; Agilent, 2014)	*h* = −12→18
*T*_min_ = 0.660, *T*_max_ = 0.880	*k* = −21→21
50539 measured reflections	*l* = −21→22

### Refinement

**Table:**

Refinement on *F*^2^	Primary atom site location: structure-invariant direct methods
Least-squares matrix: full	Secondary atom site location: difference Fourier map
*R*[*F*^2^ > 2σ(*F*^2^)] = 0.065	Hydrogen site location: mixed
*wR*(*F*^2^) = 0.160	H atoms treated by a mixture of independent and constrained refinement
*S* = 1.01	*w* = 1/[σ^2^(*F*_o_^2^) + (0.06*P*)^2^ + 6*P*] where *P* = (*F*_o_^2^ + 2*F*_c_^2^)/3
18359 reflections	(Δ/σ)_max_ = 0.001
1237 parameters	Δρ_max_ = 0.96 e Å^−^^3^
26 restraints	Δρ_min_ = −0.85 e Å^−^^3^

### Special details

**Table:**

Geometry. All esds (except the esd in the dihedral angle between two l.s. planes) are estimated using the full covariance matrix. The cell esds are taken into account individually in the estimation of esds in distances, angles and torsion angles; correlations between esds in cell parameters are only used when they are defined by crystal symmetry. An approximate (isotropic) treatment of cell esds is used for estimating esds involving l.s. planes.

### Fractional atomic coordinates and isotropic or equivalent isotropic displacement parameters (Å^2^)

**Table:**

	*x*	*y*	*z*	*U*_iso_*/*U*_eq_	Occ. (<1)
O1	−0.30049 (14)	0.81991 (14)	0.21069 (13)	0.0558 (5)	
O2	−0.24548 (15)	0.71532 (13)	0.16732 (13)	0.0563 (5)	
O3	0.14525 (13)	0.70324 (12)	0.30808 (13)	0.0499 (5)	
O4	0.41981 (13)	0.66636 (13)	0.25280 (13)	0.0541 (6)	
O5	0.73924 (19)	0.44154 (18)	0.07252 (17)	0.0814 (8)	
O6	0.81027 (19)	0.41318 (15)	0.16481 (15)	0.0741 (8)	
O7	0.67230 (13)	0.71270 (11)	0.39515 (11)	0.0431 (4)	
O8	1.11079 (14)	0.66722 (14)	0.52832 (14)	0.0590 (6)	
O9	1.07222 (16)	0.57359 (15)	0.47126 (13)	0.0639 (6)	
N1	−0.24296 (16)	0.76864 (15)	0.20575 (14)	0.0456 (6)	
N2	0.05445 (14)	0.80478 (12)	0.34212 (12)	0.0349 (5)	
H2N	0.053 (2)	0.8466 (10)	0.3671 (15)	0.042*	
N3	0.18575 (14)	0.78414 (13)	0.38582 (13)	0.0380 (5)	
H3N	0.173 (2)	0.8242 (12)	0.4129 (15)	0.046*	
N4	0.43055 (15)	0.74282 (14)	0.34333 (14)	0.0433 (5)	
N5	0.61267 (16)	0.82824 (14)	0.42129 (14)	0.0437 (5)	
H5N	0.623 (2)	0.8733 (10)	0.4382 (18)	0.052*	
N6	0.73250 (14)	0.78432 (13)	0.47369 (13)	0.0365 (5)	
H6N	0.722 (2)	0.8271 (10)	0.4972 (15)	0.044*	
N7	1.05762 (17)	0.63522 (16)	0.49814 (14)	0.0479 (6)	
N8	0.54553 (15)	0.65876 (14)	0.30550 (14)	0.0413 (5)	
H8N	0.5769 (18)	0.6786 (17)	0.3366 (14)	0.050*	
N9	0.75357 (19)	0.44901 (16)	0.13596 (17)	0.0575 (7)	
C1	0.69928 (19)	0.50461 (16)	0.17902 (17)	0.0454 (7)	
C2	0.6241 (2)	0.5329 (2)	0.1551 (2)	0.0576 (8)	
H2	0.6087	0.5183	0.1100	0.069*	
C3	0.5703 (2)	0.5832 (2)	0.19681 (19)	0.0545 (8)	
H3	0.5170	0.6023	0.1810	0.065*	
C4	0.59415 (18)	0.60564 (15)	0.26166 (16)	0.0399 (6)	
C5	0.67121 (19)	0.57639 (17)	0.28422 (17)	0.0449 (6)	
H5	0.6879	0.5916	0.3286	0.054*	
C6	0.7244 (2)	0.52534 (17)	0.24328 (18)	0.0476 (7)	
H6	0.7771	0.5051	0.2592	0.057*	
C7	0.46280 (17)	0.68813 (16)	0.29743 (16)	0.0411 (6)	
C8	0.34579 (17)	0.77955 (16)	0.33457 (16)	0.0398 (6)	
H8A	0.3335	0.7736	0.2836	0.048*	
H8B	0.3503	0.8353	0.3389	0.048*	
C9	0.26874 (17)	0.74518 (16)	0.39328 (16)	0.0392 (6)	
H9A	0.2624	0.6899	0.3876	0.047*	
H9B	0.2819	0.7492	0.4443	0.047*	
C10	0.13050 (17)	0.75938 (15)	0.34299 (15)	0.0359 (5)	
C11	−0.01842 (16)	0.79138 (15)	0.30923 (14)	0.0342 (5)	
C12	−0.02853 (18)	0.72636 (16)	0.27224 (16)	0.0400 (6)	
H12	0.0161	0.6877	0.2694	0.048*	
C13	−0.10298 (18)	0.71844 (16)	0.24008 (16)	0.0419 (6)	
H13	−0.1096	0.6745	0.2149	0.050*	
C14	−0.16814 (17)	0.77457 (16)	0.24457 (15)	0.0382 (6)	
C15	−0.16132 (18)	0.83798 (16)	0.28272 (16)	0.0412 (6)	
H15	−0.2074	0.8754	0.2868	0.049*	
C16	−0.08709 (18)	0.84628 (16)	0.31461 (15)	0.0394 (6)	
H16	−0.0820	0.8899	0.3408	0.047*	
C17	0.47607 (18)	0.76651 (17)	0.40141 (16)	0.0424 (6)	
H17A	0.5019	0.7206	0.4266	0.051*	
H17B	0.4321	0.7894	0.4399	0.051*	
C18	0.55022 (18)	0.82489 (17)	0.36954 (16)	0.0421 (6)	
H18A	0.5826	0.8104	0.3207	0.051*	
H18B	0.5238	0.8764	0.3603	0.051*	
C19	0.67255 (17)	0.77080 (15)	0.42772 (15)	0.0376 (6)	
C20	0.81048 (17)	0.74344 (15)	0.48085 (14)	0.0342 (5)	
C21	0.82524 (19)	0.66802 (16)	0.46242 (16)	0.0424 (6)	
H21	0.7800	0.6409	0.4458	0.051*	
C22	0.9062 (2)	0.63326 (16)	0.46862 (16)	0.0442 (6)	
H22	0.9170	0.5824	0.4553	0.053*	
C23	0.97117 (18)	0.67192 (16)	0.49395 (14)	0.0392 (6)	
C24	0.95747 (18)	0.74567 (16)	0.51434 (14)	0.0386 (6)	
H24	1.0023	0.7714	0.5328	0.046*	
C25	0.87735 (17)	0.78116 (15)	0.50729 (14)	0.0358 (5)	
H25	0.8674	0.8321	0.5206	0.043*	
O10A	0.0479 (3)	0.8828 (3)	0.8867 (3)	0.0689 (14)	0.5
O11A	0.0502 (7)	0.9794 (7)	0.8022 (6)	0.0689 (14)	0.5
O10B	0.0574 (3)	0.9139 (3)	0.90363 (18)	0.0515 (10)	0.5
O11B	0.0563 (6)	0.9790 (6)	0.7960 (4)	0.0515 (10)	0.5
O12	0.49661 (14)	0.78528 (13)	0.82348 (11)	0.0516 (5)	
O13	0.75401 (12)	0.75789 (11)	0.88681 (10)	0.0390 (4)	
O14	0.94784 (18)	0.54938 (14)	1.15633 (12)	0.0654 (7)	
O15	1.07221 (14)	0.52318 (12)	1.08658 (12)	0.0483 (5)	
O16	1.08082 (12)	0.76689 (11)	0.73581 (10)	0.0381 (4)	
O17	1.50576 (19)	0.61542 (18)	0.70411 (17)	0.0847 (9)	
O18	1.56917 (14)	0.68624 (14)	0.60480 (15)	0.0620 (6)	
N10	0.08998 (16)	0.93022 (17)	0.83676 (13)	0.0572 (7)	
N11	0.44803 (14)	0.86420 (13)	0.72873 (12)	0.0362 (5)	
H11N	0.4688 (19)	0.8905 (15)	0.6837 (8)	0.043*	
N12	0.58895 (15)	0.81643 (13)	0.71123 (13)	0.0392 (5)	
H12N	0.599 (2)	0.8456 (15)	0.6666 (9)	0.047*	
N13	0.81504 (13)	0.79785 (12)	0.76640 (11)	0.0336 (4)	
N14	1.03777 (14)	0.86411 (13)	0.65735 (12)	0.0361 (5)	
H14N	1.0554 (19)	0.9028 (12)	0.6211 (12)	0.043*	
N15	1.17809 (14)	0.81480 (12)	0.62862 (12)	0.0341 (4)	
H15N	1.181 (2)	0.8486 (13)	0.5873 (10)	0.041*	
N16	1.50353 (18)	0.66548 (16)	0.65167 (16)	0.0543 (6)	
N17	0.90190 (13)	0.73247 (13)	0.84589 (12)	0.0339 (4)	
H17N	0.9496 (12)	0.7446 (17)	0.8097 (12)	0.041*	
N18	0.99925 (17)	0.55484 (13)	1.09594 (13)	0.0407 (5)	
C26	1.41791 (18)	0.70288 (16)	0.64585 (17)	0.0429 (6)	
C27	1.41575 (17)	0.76631 (16)	0.59423 (15)	0.0385 (6)	
H27	1.4689	0.7852	0.5620	0.046*	
C28	1.33498 (17)	0.80203 (15)	0.59014 (14)	0.0358 (5)	
H28	1.3329	0.8463	0.5550	0.043*	
C29	1.25603 (17)	0.77435 (15)	0.63674 (14)	0.0340 (5)	
C30	1.2600 (2)	0.71000 (17)	0.68850 (19)	0.0514 (7)	
H30	1.2071	0.6901	0.7204	0.062*	
C31	1.3412 (2)	0.67522 (19)	0.6933 (2)	0.0570 (8)	
H31	1.3444	0.6320	0.7294	0.068*	
C32	1.09771 (16)	0.81194 (14)	0.67801 (14)	0.0324 (5)	
C33	0.95254 (16)	0.87103 (15)	0.70682 (15)	0.0354 (5)	
H33A	0.9242	0.9204	0.6919	0.042*	
H33B	0.9625	0.8716	0.7593	0.042*	
C34	0.89019 (16)	0.80453 (15)	0.70328 (14)	0.0339 (5)	
H34A	0.8669	0.8123	0.6553	0.041*	
H34B	0.9244	0.7558	0.7037	0.041*	
C35	0.73043 (16)	0.83373 (15)	0.75436 (14)	0.0341 (5)	
H35A	0.7427	0.8774	0.7142	0.041*	
H35B	0.7013	0.8546	0.8012	0.041*	
C36	0.66706 (18)	0.77744 (16)	0.73211 (16)	0.0403 (6)	
H36A	0.6985	0.7515	0.6891	0.048*	
H36B	0.6482	0.7375	0.7749	0.048*	
C37	0.51073 (17)	0.81871 (15)	0.76007 (15)	0.0368 (5)	
C38	0.35982 (17)	0.87685 (15)	0.75916 (14)	0.0357 (5)	
C39	0.31353 (17)	0.93099 (15)	0.71747 (14)	0.0361 (5)	
H39	0.3438	0.9566	0.6712	0.043*	
C40	0.22548 (18)	0.94750 (16)	0.74230 (15)	0.0393 (6)	
H40	0.1946	0.9835	0.7131	0.047*	
C41	0.18252 (18)	0.91135 (18)	0.80989 (16)	0.0444 (6)	
C42	0.2256 (2)	0.8569 (2)	0.85174 (17)	0.0523 (8)	
H42	0.1943	0.8314	0.8977	0.063*	
C43	0.31411 (19)	0.83923 (18)	0.82708 (16)	0.0457 (7)	
H43	0.3437	0.8019	0.8559	0.055*	
C44	0.81967 (16)	0.76260 (14)	0.83621 (14)	0.0320 (5)	
C45	0.92141 (16)	0.68865 (14)	0.91061 (14)	0.0323 (5)	
C46	0.85796 (18)	0.66579 (15)	0.97486 (15)	0.0379 (6)	
H46	0.7971	0.6798	0.9766	0.045*	
C47	0.88410 (18)	0.62279 (15)	1.03564 (15)	0.0389 (6)	
H47	0.8416	0.6082	1.0799	0.047*	
C48	0.97190 (18)	0.60118 (14)	1.03184 (14)	0.0348 (5)	
C49	1.03608 (17)	0.62206 (15)	0.96873 (14)	0.0358 (5)	
H49	1.0964	0.6061	0.9669	0.043*	
C50	1.01033 (17)	0.66647 (15)	0.90874 (14)	0.0353 (5)	
H50	1.0537	0.6823	0.8654	0.042*	
N19	0.25010 (15)	0.49825 (13)	0.23222 (13)	0.0388 (5)	
C51	−0.0401 (2)	0.47538 (19)	0.33601 (18)	0.0553 (8)	
H51A	−0.1042	0.4634	0.3455	0.083*	
H51B	−0.0290	0.5239	0.3032	0.083*	
H51C	−0.0205	0.4804	0.3839	0.083*	
C52	0.0109 (2)	0.41156 (17)	0.29828 (17)	0.0466 (7)	
H52A	−0.0015	0.4131	0.2463	0.056*	
H52B	−0.0115	0.3616	0.3258	0.056*	
C53	0.1128 (2)	0.41542 (17)	0.29455 (18)	0.0490 (7)	
H53A	0.1264	0.4087	0.3463	0.059*	
H53B	0.1417	0.3730	0.2672	0.059*	
C54	0.14972 (19)	0.49192 (16)	0.25485 (16)	0.0419 (6)	
H54A	0.1231	0.5034	0.2089	0.050*	
H54B	0.1294	0.5324	0.2882	0.050*	
C55	0.2823 (2)	0.44540 (16)	0.17047 (17)	0.0441 (6)	
H55A	0.2651	0.3918	0.1909	0.053*	
H55B	0.2502	0.4596	0.1280	0.053*	
C56	0.3813 (2)	0.44747 (18)	0.14002 (18)	0.0501 (7)	
H56A	0.4141	0.4263	0.1802	0.060*	
H56B	0.4009	0.5015	0.1242	0.060*	
C57	0.4029 (2)	0.4010 (2)	0.0735 (2)	0.0617 (9)	
H57A	0.3746	0.4255	0.0318	0.074*	
H57B	0.3773	0.3486	0.0881	0.074*	
C58	0.5029 (3)	0.3949 (2)	0.0461 (2)	0.0718 (11)	
H58A	0.5136	0.3670	0.0012	0.108*	
H58B	0.5306	0.3669	0.0859	0.108*	
H58C	0.5290	0.4466	0.0335	0.108*	
C59	0.2973 (2)	0.47440 (17)	0.29835 (17)	0.0437 (6)	
H59A	0.2862	0.4187	0.3137	0.052*	
H59B	0.3624	0.4814	0.2807	0.052*	
C60	0.2710 (2)	0.51657 (19)	0.36676 (18)	0.0514 (7)	
H60A	0.2084	0.5036	0.3901	0.062*	
H60B	0.2747	0.5728	0.3515	0.062*	
C61	0.3314 (3)	0.4954 (2)	0.4237 (2)	0.0631 (9)	
H61A	0.3282	0.4389	0.4372	0.076*	
H61B	0.3936	0.5084	0.3993	0.076*	
C62	0.3110 (4)	0.5334 (3)	0.4944 (2)	0.0875 (14)	
H62A	0.3552	0.5178	0.5264	0.131*	
H62B	0.2512	0.5178	0.5214	0.131*	
H62C	0.3131	0.5894	0.4820	0.131*	
C63	0.27396 (19)	0.58148 (15)	0.20295 (16)	0.0415 (6)	
H63A	0.2445	0.6146	0.2410	0.050*	
H63B	0.3393	0.5879	0.1989	0.050*	
C64	0.2492 (2)	0.61080 (17)	0.12773 (18)	0.0501 (7)	
H64A	0.2855	0.5840	0.0875	0.060*	
H64B	0.1855	0.5991	0.1285	0.060*	
C65	0.2646 (3)	0.69612 (18)	0.1108 (2)	0.0595 (8)	
H65A	0.3254	0.7080	0.1189	0.071*	
H65B	0.2215	0.7226	0.1472	0.071*	
C66	0.2553 (3)	0.7282 (2)	0.0323 (2)	0.0682 (10)	
H66A	0.2710	0.7831	0.0246	0.102*	
H66B	0.1935	0.7219	0.0254	0.102*	
H66C	0.2954	0.7007	−0.0043	0.102*	
N20	0.72892 (17)	0.93841 (15)	1.01240 (14)	0.0479 (6)	
C67	0.8071 (2)	0.93305 (19)	1.05475 (17)	0.0503 (7)	
H67A	0.8379	0.9837	1.0458	0.060*	
H67B	0.7829	0.9238	1.1094	0.060*	
C68	0.8757 (2)	0.8720 (2)	1.03507 (18)	0.0536 (8)	
H68A	0.8963	0.8771	0.9798	0.064*	
H68B	0.8478	0.8205	1.0502	0.064*	
C69	0.9551 (2)	0.8784 (2)	1.07384 (18)	0.0549 (8)	
H69A	0.9879	0.9267	1.0529	0.066*	
H69B	0.9334	0.8816	1.1282	0.066*	
C70	1.0183 (2)	0.8117 (2)	1.0650 (2)	0.0621 (9)	
H70A	1.0689	0.8195	1.0903	0.093*	
H70B	1.0404	0.8082	1.0113	0.093*	
H70C	0.9870	0.7638	1.0876	0.093*	
C71	0.6633 (2)	0.99719 (18)	1.04517 (18)	0.0527 (7)	
H71A	0.6200	1.0093	1.0107	0.063*	
H71B	0.6964	1.0452	1.0470	0.063*	
C72	0.6114 (2)	0.97207 (18)	1.12398 (16)	0.0494 (7)	
H72A	0.6021	0.9156	1.1297	0.059*	
H72B	0.6472	0.9842	1.1619	0.059*	
C73	0.5216 (2)	1.0113 (2)	1.13913 (19)	0.0559 (8)	
H73A	0.4876	1.0027	1.0989	0.067*	
H73B	0.5310	1.0675	1.1376	0.067*	
C74	0.4670 (2)	0.9813 (2)	1.2154 (2)	0.0633 (9)	
H74A	0.4089	1.0071	1.2223	0.095*	
H74B	0.4991	0.9920	1.2556	0.095*	
H74C	0.4580	0.9256	1.2173	0.095*	
C75	0.6859 (2)	0.86013 (17)	1.01881 (17)	0.0467 (7)	
H75A	0.7288	0.8256	0.9911	0.056*	
H75B	0.6750	0.8389	1.0726	0.056*	
C76	0.5992 (2)	0.85802 (19)	0.98967 (18)	0.0509 (7)	
H76A	0.6114	0.8659	0.9339	0.061*	
H76B	0.5603	0.9004	1.0081	0.061*	
C77	0.5512 (2)	0.7811 (2)	1.0158 (2)	0.0593 (8)	
H77A	0.5044	0.7754	0.9851	0.071*	
H77B	0.5946	0.7387	1.0071	0.071*	
C78	0.5094 (3)	0.7741 (3)	1.0970 (3)	0.0975 (16)	
H78A	0.4813	0.7232	1.1113	0.146*	
H78B	0.4640	0.8141	1.1055	0.146*	
H78C	0.5554	0.7803	1.1276	0.146*	
C79	0.7611 (2)	0.96494 (19)	0.92815 (18)	0.0598 (9)	
H79A	0.7876	0.9200	0.9036	0.072*	0.5
H79B	0.7080	0.9806	0.9055	0.072*	0.5
H79C	0.8093	0.9301	0.9082	0.072*	0.5
H79D	0.7109	0.9592	0.9010	0.072*	0.5
C80A	0.8284 (6)	1.0305 (5)	0.9088 (4)	0.067 (3)	0.5
H80A	0.8051	1.0748	0.9359	0.080*	0.5
H80B	0.8849	1.0138	0.9256	0.080*	0.5
C81A	0.8466 (4)	1.0550 (4)	0.8248 (3)	0.0606 (12)	0.5
H81A	0.8875	1.1000	0.8159	0.073*	0.5
H81B	0.8796	1.0127	0.8000	0.073*	0.5
C82A	0.7693 (6)	1.0761 (7)	0.7843 (6)	0.090 (3)	0.5
H82A	0.7894	1.0765	0.7298	0.136*	0.5
H82B	0.7473	1.1274	0.7956	0.136*	0.5
H82C	0.7211	1.0382	0.8013	0.136*	0.5
C80B	0.7954 (6)	1.0474 (3)	0.9106 (4)	0.067 (3)	0.5
H80C	0.7636	1.0778	0.9500	0.080*	0.5
H80D	0.8594	1.0472	0.9148	0.080*	0.5
C81B	0.7864 (5)	1.0888 (4)	0.8344 (3)	0.0606 (12)	0.5
H81C	0.7231	1.1037	0.8355	0.073*	0.5
H81D	0.8224	1.1367	0.8259	0.073*	0.5
C82B	0.8152 (8)	1.0424 (6)	0.7690 (5)	0.090 (3)	0.5
H82D	0.8166	1.0756	0.7216	0.136*	0.5
H82E	0.7729	1.0000	0.7716	0.136*	0.5
H82F	0.8750	1.0215	0.7711	0.136*	0.5
P1	0.60995 (4)	0.97408 (3)	0.55144 (3)	0.02832 (13)	
O19	0.51551 (11)	0.95514 (10)	0.59024 (10)	0.0354 (4)	
O20	0.63474 (11)	1.05356 (10)	0.57479 (10)	0.0344 (4)	
H20O	0.6934 (8)	1.0670 (18)	0.5542 (17)	0.052*	
O21	0.61479 (11)	0.98453 (10)	0.46370 (10)	0.0333 (4)	
H21O	0.5645 (13)	1.0068 (17)	0.4480 (17)	0.050*	
O22	0.67758 (11)	0.91334 (9)	0.56726 (10)	0.0327 (4)	
P2	0.87010 (4)	1.03192 (3)	0.53218 (3)	0.02841 (13)	
O23	0.82990 (11)	0.96052 (10)	0.58748 (10)	0.0338 (4)	
H23O	0.7761 (11)	0.9427 (18)	0.5783 (18)	0.051*	
O24	0.79916 (11)	1.09098 (9)	0.52118 (10)	0.0335 (4)	
O25	0.90787 (11)	1.00351 (10)	0.45455 (10)	0.0332 (4)	
H25O	0.9644 (9)	0.9823 (17)	0.4529 (18)	0.050*	
O26	0.94470 (11)	1.06344 (10)	0.56654 (10)	0.0363 (4)	

### Atomic displacement parameters (Å^2^)

**Table:**

	*U*^11^	*U*^22^	*U*^33^	*U*^12^	*U*^13^	*U*^23^
O1	0.0425 (11)	0.0716 (15)	0.0544 (13)	0.0122 (11)	−0.0132 (9)	−0.0063 (11)
O2	0.0513 (13)	0.0562 (13)	0.0662 (14)	−0.0077 (10)	−0.0194 (11)	−0.0112 (11)
O3	0.0413 (11)	0.0482 (12)	0.0658 (13)	0.0115 (9)	−0.0135 (9)	−0.0266 (10)
O4	0.0356 (10)	0.0673 (14)	0.0682 (14)	0.0052 (9)	−0.0164 (10)	−0.0359 (11)
O5	0.0706 (17)	0.097 (2)	0.0833 (19)	0.0183 (15)	−0.0055 (14)	−0.0533 (17)
O6	0.0820 (18)	0.0580 (15)	0.0704 (16)	0.0327 (13)	0.0133 (14)	−0.0017 (12)
O7	0.0398 (10)	0.0426 (10)	0.0526 (11)	0.0068 (8)	−0.0151 (8)	−0.0203 (9)
O8	0.0359 (11)	0.0642 (14)	0.0735 (15)	0.0015 (10)	−0.0116 (10)	0.0110 (12)
O9	0.0655 (15)	0.0680 (15)	0.0571 (14)	0.0338 (12)	−0.0088 (11)	−0.0101 (12)
N1	0.0373 (12)	0.0530 (15)	0.0434 (13)	−0.0048 (11)	−0.0037 (10)	0.0035 (11)
N2	0.0308 (11)	0.0336 (11)	0.0405 (11)	0.0023 (9)	−0.0043 (9)	−0.0076 (9)
N3	0.0324 (11)	0.0358 (11)	0.0468 (13)	0.0048 (9)	−0.0065 (9)	−0.0107 (10)
N4	0.0320 (11)	0.0535 (14)	0.0479 (13)	0.0067 (10)	−0.0087 (10)	−0.0200 (11)
N5	0.0424 (13)	0.0403 (13)	0.0554 (14)	0.0086 (10)	−0.0197 (11)	−0.0189 (11)
N6	0.0356 (11)	0.0352 (11)	0.0418 (12)	0.0043 (9)	−0.0092 (9)	−0.0141 (9)
N7	0.0406 (13)	0.0557 (15)	0.0413 (13)	0.0100 (11)	0.0003 (10)	0.0085 (11)
N8	0.0321 (11)	0.0448 (13)	0.0506 (13)	0.0014 (9)	−0.0085 (10)	−0.0194 (11)
N9	0.0563 (16)	0.0452 (15)	0.0639 (18)	0.0001 (13)	0.0136 (14)	−0.0143 (13)
C1	0.0410 (15)	0.0352 (14)	0.0548 (17)	0.0003 (11)	0.0081 (12)	−0.0088 (12)
C2	0.0508 (18)	0.065 (2)	0.061 (2)	0.0011 (15)	−0.0077 (15)	−0.0305 (17)
C3	0.0390 (16)	0.065 (2)	0.066 (2)	0.0095 (14)	−0.0127 (14)	−0.0300 (16)
C4	0.0345 (13)	0.0362 (14)	0.0482 (15)	−0.0044 (11)	−0.0007 (11)	−0.0106 (11)
C5	0.0441 (15)	0.0435 (15)	0.0474 (16)	0.0040 (12)	−0.0064 (12)	−0.0092 (12)
C6	0.0453 (16)	0.0409 (15)	0.0534 (17)	0.0081 (12)	−0.0018 (13)	−0.0028 (13)
C7	0.0296 (13)	0.0452 (15)	0.0498 (15)	−0.0024 (11)	−0.0041 (11)	−0.0156 (12)
C8	0.0336 (13)	0.0435 (15)	0.0445 (15)	0.0042 (11)	−0.0086 (11)	−0.0127 (12)
C9	0.0337 (13)	0.0360 (13)	0.0496 (15)	0.0034 (11)	−0.0102 (11)	−0.0078 (11)
C10	0.0298 (12)	0.0346 (13)	0.0410 (14)	0.0020 (10)	−0.0006 (10)	−0.0036 (11)
C11	0.0313 (12)	0.0351 (13)	0.0333 (12)	−0.0007 (10)	−0.0004 (10)	0.0005 (10)
C12	0.0361 (14)	0.0365 (14)	0.0465 (15)	0.0034 (11)	−0.0038 (11)	−0.0064 (11)
C13	0.0390 (14)	0.0397 (14)	0.0459 (15)	−0.0029 (11)	−0.0044 (11)	−0.0051 (12)
C14	0.0312 (13)	0.0439 (15)	0.0372 (13)	−0.0040 (11)	−0.0030 (10)	0.0017 (11)
C15	0.0369 (14)	0.0413 (15)	0.0434 (14)	0.0059 (11)	−0.0036 (11)	−0.0017 (12)
C16	0.0396 (14)	0.0371 (14)	0.0411 (14)	0.0034 (11)	−0.0052 (11)	−0.0057 (11)
C17	0.0359 (14)	0.0525 (16)	0.0409 (14)	0.0088 (12)	−0.0067 (11)	−0.0161 (12)
C18	0.0399 (14)	0.0448 (15)	0.0456 (15)	0.0082 (12)	−0.0134 (12)	−0.0140 (12)
C19	0.0330 (13)	0.0390 (14)	0.0424 (14)	0.0020 (10)	−0.0070 (11)	−0.0103 (11)
C20	0.0367 (13)	0.0355 (13)	0.0298 (12)	0.0032 (10)	−0.0041 (10)	−0.0045 (10)
C21	0.0446 (15)	0.0377 (14)	0.0477 (15)	0.0017 (12)	−0.0129 (12)	−0.0098 (12)
C22	0.0515 (16)	0.0366 (14)	0.0453 (15)	0.0098 (12)	−0.0086 (12)	−0.0086 (12)
C23	0.0377 (14)	0.0450 (15)	0.0316 (12)	0.0087 (11)	−0.0015 (10)	0.0019 (11)
C24	0.0364 (13)	0.0458 (15)	0.0337 (13)	−0.0031 (11)	−0.0081 (10)	−0.0012 (11)
C25	0.0393 (14)	0.0356 (13)	0.0335 (12)	0.0027 (10)	−0.0070 (10)	−0.0068 (10)
O10A	0.043 (2)	0.098 (3)	0.065 (3)	−0.001 (2)	−0.002 (2)	−0.017 (3)
O11A	0.043 (2)	0.098 (3)	0.065 (3)	−0.001 (2)	−0.002 (2)	−0.017 (3)
O10B	0.038 (2)	0.058 (2)	0.056 (2)	0.0010 (16)	0.0051 (16)	−0.0169 (18)
O11B	0.038 (2)	0.058 (2)	0.056 (2)	0.0010 (16)	0.0051 (16)	−0.0169 (18)
O12	0.0471 (11)	0.0621 (13)	0.0424 (11)	0.0037 (10)	−0.0108 (9)	0.0127 (10)
O13	0.0315 (9)	0.0488 (11)	0.0337 (9)	0.0046 (8)	−0.0006 (7)	0.0005 (8)
O14	0.0855 (17)	0.0689 (15)	0.0343 (11)	0.0219 (13)	−0.0013 (11)	0.0100 (10)
O15	0.0469 (12)	0.0517 (12)	0.0498 (11)	0.0020 (9)	−0.0223 (9)	0.0021 (9)
O16	0.0326 (9)	0.0421 (10)	0.0364 (9)	0.0011 (7)	−0.0039 (7)	0.0060 (8)
O17	0.0686 (17)	0.089 (2)	0.087 (2)	0.0363 (15)	−0.0099 (14)	0.0231 (16)
O18	0.0383 (12)	0.0599 (14)	0.0859 (17)	0.0104 (10)	−0.0055 (11)	−0.0102 (12)
N10	0.0393 (14)	0.084 (2)	0.0456 (15)	−0.0057 (14)	0.0019 (11)	−0.0091 (14)
N11	0.0310 (11)	0.0421 (12)	0.0339 (11)	−0.0014 (9)	−0.0063 (8)	0.0042 (9)
N12	0.0318 (11)	0.0423 (12)	0.0429 (12)	0.0022 (9)	−0.0107 (9)	0.0048 (10)
N13	0.0271 (10)	0.0404 (11)	0.0319 (10)	0.0013 (8)	−0.0045 (8)	0.0007 (9)
N14	0.0322 (11)	0.0359 (11)	0.0366 (11)	0.0002 (9)	−0.0017 (9)	0.0057 (9)
N15	0.0309 (10)	0.0356 (11)	0.0338 (11)	−0.0003 (9)	−0.0045 (8)	0.0029 (9)
N16	0.0477 (15)	0.0517 (15)	0.0648 (17)	0.0146 (12)	−0.0133 (13)	−0.0084 (13)
N17	0.0272 (10)	0.0413 (12)	0.0312 (10)	0.0012 (9)	−0.0034 (8)	0.0014 (9)
N18	0.0530 (14)	0.0345 (11)	0.0365 (12)	−0.0021 (10)	−0.0141 (10)	−0.0017 (9)
C26	0.0362 (14)	0.0415 (15)	0.0515 (16)	0.0082 (11)	−0.0081 (12)	−0.0073 (12)
C27	0.0315 (13)	0.0465 (15)	0.0378 (13)	−0.0002 (11)	−0.0053 (10)	−0.0070 (11)
C28	0.0354 (13)	0.0394 (14)	0.0328 (12)	−0.0008 (11)	−0.0088 (10)	−0.0001 (10)
C29	0.0331 (13)	0.0338 (13)	0.0358 (13)	0.0010 (10)	−0.0069 (10)	−0.0055 (10)
C30	0.0400 (15)	0.0429 (16)	0.0634 (19)	0.0043 (12)	0.0020 (13)	0.0111 (14)
C31	0.0521 (18)	0.0437 (17)	0.068 (2)	0.0091 (14)	−0.0049 (15)	0.0162 (15)
C32	0.0301 (12)	0.0344 (12)	0.0332 (12)	−0.0031 (10)	−0.0075 (10)	−0.0022 (10)
C33	0.0323 (13)	0.0349 (13)	0.0371 (13)	0.0036 (10)	−0.0037 (10)	−0.0002 (10)
C34	0.0320 (12)	0.0395 (13)	0.0297 (12)	0.0044 (10)	−0.0064 (9)	−0.0005 (10)
C35	0.0316 (12)	0.0351 (13)	0.0348 (12)	0.0045 (10)	−0.0059 (10)	−0.0012 (10)
C36	0.0364 (14)	0.0374 (14)	0.0491 (15)	0.0041 (11)	−0.0147 (12)	−0.0017 (12)
C37	0.0339 (13)	0.0375 (13)	0.0398 (14)	−0.0028 (10)	−0.0111 (10)	−0.0004 (11)
C38	0.0323 (13)	0.0411 (14)	0.0347 (13)	−0.0048 (10)	−0.0081 (10)	−0.0045 (10)
C39	0.0339 (13)	0.0400 (14)	0.0332 (12)	−0.0028 (10)	−0.0036 (10)	−0.0018 (10)
C40	0.0348 (13)	0.0419 (14)	0.0430 (14)	−0.0008 (11)	−0.0099 (11)	−0.0074 (11)
C41	0.0328 (14)	0.0601 (18)	0.0411 (14)	−0.0050 (12)	−0.0055 (11)	−0.0100 (13)
C42	0.0404 (16)	0.076 (2)	0.0365 (14)	−0.0116 (15)	−0.0004 (12)	0.0055 (14)
C43	0.0394 (15)	0.0570 (18)	0.0391 (14)	−0.0046 (13)	−0.0094 (11)	0.0066 (13)
C44	0.0304 (12)	0.0325 (12)	0.0338 (12)	−0.0019 (10)	−0.0065 (10)	−0.0050 (10)
C45	0.0329 (12)	0.0317 (12)	0.0331 (12)	−0.0006 (10)	−0.0081 (10)	−0.0035 (10)
C46	0.0336 (13)	0.0390 (14)	0.0393 (14)	0.0003 (11)	−0.0043 (10)	0.0002 (11)
C47	0.0417 (14)	0.0373 (14)	0.0354 (13)	−0.0029 (11)	−0.0027 (11)	0.0006 (11)
C48	0.0437 (14)	0.0298 (12)	0.0331 (12)	−0.0016 (10)	−0.0128 (10)	−0.0031 (10)
C49	0.0328 (13)	0.0392 (13)	0.0373 (13)	0.0016 (10)	−0.0108 (10)	−0.0059 (11)
C50	0.0343 (13)	0.0400 (14)	0.0310 (12)	−0.0011 (10)	−0.0048 (10)	−0.0028 (10)
N19	0.0423 (12)	0.0335 (11)	0.0428 (12)	−0.0016 (9)	−0.0144 (10)	−0.0027 (9)
C51	0.062 (2)	0.0517 (18)	0.0486 (17)	0.0078 (15)	−0.0042 (14)	0.0006 (14)
C52	0.0503 (17)	0.0413 (15)	0.0474 (16)	−0.0070 (13)	−0.0065 (13)	−0.0032 (12)
C53	0.0538 (17)	0.0398 (15)	0.0518 (17)	0.0016 (13)	−0.0064 (14)	−0.0022 (13)
C54	0.0422 (15)	0.0417 (15)	0.0439 (15)	0.0008 (12)	−0.0125 (12)	−0.0057 (12)
C55	0.0520 (17)	0.0333 (14)	0.0476 (16)	−0.0013 (12)	−0.0117 (13)	−0.0025 (12)
C56	0.0531 (18)	0.0451 (16)	0.0514 (17)	−0.0021 (13)	−0.0068 (14)	−0.0063 (13)
C57	0.072 (2)	0.0525 (19)	0.0550 (19)	−0.0169 (17)	0.0095 (16)	−0.0122 (15)
C58	0.078 (3)	0.055 (2)	0.074 (2)	−0.0113 (18)	0.017 (2)	−0.0163 (18)
C59	0.0438 (15)	0.0395 (14)	0.0500 (16)	0.0046 (12)	−0.0144 (12)	−0.0051 (12)
C60	0.0599 (19)	0.0481 (17)	0.0492 (17)	0.0090 (14)	−0.0157 (14)	−0.0102 (13)
C61	0.074 (2)	0.068 (2)	0.0519 (19)	0.0132 (18)	−0.0228 (17)	−0.0063 (16)
C62	0.121 (4)	0.084 (3)	0.069 (3)	0.029 (3)	−0.046 (3)	−0.015 (2)
C63	0.0415 (14)	0.0342 (13)	0.0506 (16)	−0.0023 (11)	−0.0120 (12)	−0.0066 (12)
C64	0.0552 (18)	0.0417 (16)	0.0541 (17)	−0.0067 (13)	−0.0153 (14)	0.0022 (13)
C65	0.074 (2)	0.0414 (17)	0.065 (2)	−0.0048 (15)	−0.0199 (17)	0.0006 (15)
C66	0.091 (3)	0.0472 (19)	0.063 (2)	0.0067 (18)	−0.0140 (19)	0.0076 (16)
N20	0.0531 (15)	0.0519 (14)	0.0406 (13)	−0.0053 (11)	−0.0087 (11)	−0.0129 (11)
C67	0.0523 (17)	0.0582 (18)	0.0408 (15)	−0.0126 (14)	−0.0053 (13)	−0.0096 (13)
C68	0.0475 (17)	0.063 (2)	0.0480 (17)	−0.0132 (15)	0.0014 (13)	−0.0117 (15)
C69	0.0541 (18)	0.061 (2)	0.0472 (17)	−0.0120 (15)	−0.0005 (14)	−0.0053 (14)
C70	0.057 (2)	0.067 (2)	0.060 (2)	−0.0052 (17)	−0.0065 (16)	−0.0035 (17)
C71	0.067 (2)	0.0455 (16)	0.0501 (17)	−0.0005 (14)	−0.0205 (15)	−0.0084 (13)
C72	0.0644 (19)	0.0467 (16)	0.0410 (15)	0.0068 (14)	−0.0159 (14)	−0.0120 (13)
C73	0.0595 (19)	0.0573 (19)	0.0587 (19)	0.0093 (15)	−0.0258 (16)	−0.0168 (15)
C74	0.060 (2)	0.063 (2)	0.069 (2)	0.0039 (16)	−0.0069 (17)	−0.0198 (18)
C75	0.0505 (17)	0.0475 (16)	0.0407 (15)	−0.0061 (13)	−0.0014 (12)	−0.0088 (12)
C76	0.0552 (18)	0.0539 (18)	0.0446 (16)	−0.0024 (14)	−0.0098 (13)	−0.0079 (13)
C77	0.0534 (19)	0.0545 (19)	0.071 (2)	−0.0091 (15)	−0.0126 (16)	−0.0060 (16)
C78	0.082 (3)	0.115 (4)	0.088 (3)	−0.044 (3)	−0.011 (2)	0.017 (3)
C79	0.070 (2)	0.068 (2)	0.0420 (16)	−0.0177 (18)	−0.0090 (15)	−0.0090 (15)
C80A	0.053 (6)	0.093 (4)	0.052 (2)	−0.034 (4)	−0.006 (3)	−0.003 (3)
C81A	0.058 (3)	0.063 (3)	0.056 (3)	−0.008 (2)	0.006 (2)	−0.006 (2)
C82A	0.093 (7)	0.100 (8)	0.070 (4)	−0.024 (5)	−0.010 (5)	0.025 (4)
C80B	0.053 (6)	0.093 (4)	0.052 (2)	−0.034 (4)	−0.006 (3)	−0.003 (3)
C81B	0.058 (3)	0.063 (3)	0.056 (3)	−0.008 (2)	0.006 (2)	−0.006 (2)
C82B	0.093 (7)	0.100 (8)	0.070 (4)	−0.024 (5)	−0.010 (5)	0.025 (4)
P1	0.0237 (3)	0.0299 (3)	0.0312 (3)	−0.0007 (2)	−0.0050 (2)	−0.0017 (2)
O19	0.0295 (9)	0.0420 (10)	0.0336 (9)	−0.0027 (7)	−0.0072 (7)	0.0043 (7)
O20	0.0240 (8)	0.0358 (9)	0.0435 (10)	0.0032 (7)	−0.0043 (7)	−0.0080 (7)
O21	0.0270 (8)	0.0365 (9)	0.0352 (9)	0.0010 (7)	−0.0034 (7)	−0.0015 (7)
O22	0.0303 (8)	0.0308 (9)	0.0375 (9)	−0.0013 (7)	−0.0079 (7)	−0.0027 (7)
P2	0.0229 (3)	0.0267 (3)	0.0354 (3)	0.0013 (2)	−0.0054 (2)	−0.0021 (2)
O23	0.0261 (8)	0.0337 (9)	0.0417 (9)	0.0002 (7)	−0.0101 (7)	0.0030 (7)
O24	0.0271 (8)	0.0296 (8)	0.0425 (9)	0.0008 (7)	−0.0043 (7)	−0.0006 (7)
O25	0.0258 (8)	0.0340 (9)	0.0405 (9)	0.0022 (7)	−0.0082 (7)	−0.0032 (7)
O26	0.0292 (9)	0.0341 (9)	0.0475 (10)	0.0042 (7)	−0.0074 (7)	−0.0117 (8)

### Geometric parameters (Å, º)

**Table:**

O1—N1	1.242 (3)	C46—H46	0.9500
O2—N1	1.226 (3)	C47—C48	1.374 (4)
O3—C10	1.220 (3)	C47—H47	0.9500
O4—C7	1.219 (3)	C48—C49	1.385 (4)
O5—N9	1.228 (4)	C49—C50	1.377 (4)
O6—N9	1.217 (4)	C49—H49	0.9500
O7—C19	1.228 (3)	C50—H50	0.9500
O8—N7	1.225 (3)	N19—C54	1.511 (4)
O9—N7	1.230 (3)	N19—C63	1.518 (3)
N1—C14	1.450 (4)	N19—C59	1.521 (3)
N2—C11	1.382 (3)	N19—C55	1.536 (4)
N2—C10	1.394 (3)	C51—C52	1.504 (4)
N2—H2N	0.898 (5)	C51—H51A	0.9800
N3—C10	1.345 (3)	C51—H51B	0.9800
N3—C9	1.445 (3)	C51—H51C	0.9800
N3—H3N	0.897 (5)	C52—C53	1.541 (4)
N4—C7	1.363 (3)	C52—H52A	0.9900
N4—C17	1.455 (3)	C52—H52B	0.9900
N4—C8	1.457 (3)	C53—C54	1.512 (4)
N5—C19	1.354 (3)	C53—H53A	0.9900
N5—C18	1.454 (3)	C53—H53B	0.9900
N5—H5N	0.899 (5)	C54—H54A	0.9900
N6—C19	1.378 (3)	C54—H54B	0.9900
N6—C20	1.394 (3)	C55—C56	1.509 (4)
N6—H6N	0.897 (5)	C55—H55A	0.9900
N7—C23	1.464 (3)	C55—H55B	0.9900
N8—C7	1.377 (4)	C56—C57	1.510 (4)
N8—C4	1.403 (3)	C56—H56A	0.9900
N8—H8N	0.898 (5)	C56—H56B	0.9900
N9—C1	1.465 (4)	C57—C58	1.520 (5)
C1—C2	1.361 (5)	C57—H57A	0.9900
C1—C6	1.373 (4)	C57—H57B	0.9900
C2—C3	1.385 (4)	C58—H58A	0.9800
C2—H2	0.9500	C58—H58B	0.9800
C3—C4	1.386 (4)	C58—H58C	0.9800
C3—H3	0.9500	C59—C60	1.501 (4)
C4—C5	1.381 (4)	C59—H59A	0.9900
C5—C6	1.382 (4)	C59—H59B	0.9900
C5—H5	0.9500	C60—C61	1.512 (4)
C6—H6	0.9500	C60—H60A	0.9900
C8—C9	1.518 (4)	C60—H60B	0.9900
C8—H8A	0.9900	C61—C62	1.488 (5)
C8—H8B	0.9900	C61—H61A	0.9900
C9—H9A	0.9900	C61—H61B	0.9900
C9—H9B	0.9900	C62—H62A	0.9800
C11—C12	1.402 (4)	C62—H62B	0.9800
C11—C16	1.405 (4)	C62—H62C	0.9800
C12—C13	1.377 (4)	C63—C64	1.512 (4)
C12—H12	0.9500	C63—H63A	0.9900
C13—C14	1.382 (4)	C63—H63B	0.9900
C13—H13	0.9500	C64—C65	1.501 (4)
C14—C15	1.381 (4)	C64—H64A	0.9900
C15—C16	1.372 (4)	C64—H64B	0.9900
C15—H15	0.9500	C65—C66	1.501 (5)
C16—H16	0.9500	C65—H65A	0.9900
C17—C18	1.523 (4)	C65—H65B	0.9900
C17—H17A	0.9900	C66—H66A	0.9800
C17—H17B	0.9900	C66—H66B	0.9800
C18—H18A	0.9900	C66—H66C	0.9800
C18—H18B	0.9900	N20—C75	1.507 (4)
C20—C21	1.398 (4)	N20—C71	1.515 (4)
C20—C25	1.399 (4)	N20—C67	1.522 (4)
C21—C22	1.382 (4)	N20—C79	1.541 (4)
C21—H21	0.9500	C67—C68	1.507 (5)
C22—C23	1.374 (4)	C67—H67A	0.9900
C22—H22	0.9500	C67—H67B	0.9900
C23—C24	1.380 (4)	C68—C69	1.511 (5)
C24—C25	1.379 (4)	C68—H68A	0.9900
C24—H24	0.9500	C68—H68B	0.9900
C25—H25	0.9500	C69—C70	1.504 (5)
O10A—N10	1.250 (3)	C69—H69A	0.9900
O11A—N10	1.224 (4)	C69—H69B	0.9900
O10B—N10	1.230 (3)	C70—H70A	0.9800
O11B—N10	1.232 (3)	C70—H70B	0.9800
O12—C37	1.216 (3)	C70—H70C	0.9800
O13—C44	1.227 (3)	C71—C72	1.527 (4)
O14—N18	1.223 (3)	C71—H71A	0.9900
O15—N18	1.225 (3)	C71—H71B	0.9900
O16—C32	1.231 (3)	C72—C73	1.513 (4)
O17—N16	1.224 (4)	C72—H72A	0.9900
O18—N16	1.222 (4)	C72—H72B	0.9900
N10—C41	1.451 (4)	C73—C74	1.524 (5)
N11—C38	1.381 (3)	C73—H73A	0.9900
N11—C37	1.391 (3)	C73—H73B	0.9900
N11—H11N	0.899 (5)	C74—H74A	0.9800
N12—C37	1.354 (3)	C74—H74B	0.9800
N12—C36	1.448 (3)	C74—H74C	0.9800
N12—H12N	0.897 (5)	C75—C76	1.506 (4)
N13—C44	1.359 (3)	C75—H75A	0.9900
N13—C34	1.463 (3)	C75—H75B	0.9900
N13—C35	1.463 (3)	C76—C77	1.526 (4)
N14—C32	1.350 (3)	C76—H76A	0.9900
N14—C33	1.451 (3)	C76—H76B	0.9900
N14—H14N	0.898 (5)	C77—C78	1.489 (6)
N15—C32	1.379 (3)	C77—H77A	0.9900
N15—C29	1.393 (3)	C77—H77B	0.9900
N15—H15N	0.897 (5)	C78—H78A	0.9800
N16—C26	1.463 (4)	C78—H78B	0.9800
N17—C44	1.382 (3)	C78—H78C	0.9800
N17—C45	1.404 (3)	C79—C80A	1.516 (4)
N17—H17N	0.898 (5)	C79—C80B	1.519 (4)
N18—C48	1.461 (3)	C79—H79A	0.9900
C26—C27	1.374 (4)	C79—H79B	0.9900
C26—C31	1.379 (4)	C79—H79C	0.9900
C27—C28	1.379 (4)	C79—H79D	0.9900
C27—H27	0.9500	C80A—C81A	1.514 (4)
C28—C29	1.399 (4)	C80A—H80A	0.9900
C28—H28	0.9500	C80A—H80B	0.9900
C29—C30	1.391 (4)	C81A—C82A	1.513 (4)
C30—C31	1.381 (4)	C81A—H81A	0.9900
C30—H30	0.9500	C81A—H81B	0.9900
C31—H31	0.9500	C82A—H82A	0.9800
C33—C34	1.523 (4)	C82A—H82B	0.9800
C33—H33A	0.9900	C82A—H82C	0.9800
C33—H33B	0.9900	C80B—C81B	1.515 (4)
C34—H34A	0.9900	C80B—H80C	0.9900
C34—H34B	0.9900	C80B—H80D	0.9900
C35—C36	1.519 (4)	C81B—C82B	1.506 (4)
C35—H35A	0.9900	C81B—H81C	0.9900
C35—H35B	0.9900	C81B—H81D	0.9900
C36—H36A	0.9900	C82B—H82D	0.9800
C36—H36B	0.9900	C82B—H82E	0.9800
C38—C43	1.402 (4)	C82B—H82F	0.9800
C38—C39	1.405 (4)	P1—O22	1.5071 (17)
C39—C40	1.372 (4)	P1—O19	1.5072 (17)
C39—H39	0.9500	P1—O20	1.5632 (18)
C40—C41	1.374 (4)	P1—O21	1.5695 (18)
C40—H40	0.9500	O20—H20O	0.928 (5)
C41—C42	1.383 (4)	O21—H21O	0.928 (5)
C42—C43	1.382 (4)	P2—O24	1.5054 (17)
C42—H42	0.9500	P2—O26	1.5226 (18)
C43—H43	0.9500	P2—O25	1.5531 (18)
C45—C50	1.397 (4)	P2—O23	1.5705 (18)
C45—C46	1.400 (4)	O23—H23O	0.927 (5)
C46—C47	1.379 (4)	O25—H25O	0.928 (5)
			
O2—N1—O1	122.6 (3)	C52—C51—H51B	109.5
O2—N1—C14	119.2 (2)	H51A—C51—H51B	109.5
O1—N1—C14	118.1 (3)	C52—C51—H51C	109.5
C11—N2—C10	127.4 (2)	H51A—C51—H51C	109.5
C11—N2—H2N	118 (2)	H51B—C51—H51C	109.5
C10—N2—H2N	115 (2)	C51—C52—C53	114.4 (3)
C10—N3—C9	121.4 (2)	C51—C52—H52A	108.7
C10—N3—H3N	123 (2)	C53—C52—H52A	108.7
C9—N3—H3N	116 (2)	C51—C52—H52B	108.7
C7—N4—C17	123.5 (2)	C53—C52—H52B	108.7
C7—N4—C8	118.8 (2)	H52A—C52—H52B	107.6
C17—N4—C8	117.8 (2)	C54—C53—C52	110.6 (2)
C19—N5—C18	119.6 (2)	C54—C53—H53A	109.5
C19—N5—H5N	118 (2)	C52—C53—H53A	109.5
C18—N5—H5N	120 (2)	C54—C53—H53B	109.5
C19—N6—C20	126.8 (2)	C52—C53—H53B	109.5
C19—N6—H6N	115 (2)	H53A—C53—H53B	108.1
C20—N6—H6N	118 (2)	N19—C54—C53	116.7 (2)
O8—N7—O9	123.9 (3)	N19—C54—H54A	108.1
O8—N7—C23	118.4 (3)	C53—C54—H54A	108.1
O9—N7—C23	117.7 (3)	N19—C54—H54B	108.1
C7—N8—C4	126.1 (2)	C53—C54—H54B	108.1
C7—N8—H8N	120 (2)	H54A—C54—H54B	107.3
C4—N8—H8N	113 (2)	C56—C55—N19	115.7 (2)
O6—N9—O5	123.8 (3)	C56—C55—H55A	108.3
O6—N9—C1	118.4 (3)	N19—C55—H55A	108.3
O5—N9—C1	117.8 (3)	C56—C55—H55B	108.3
C2—C1—C6	121.9 (3)	N19—C55—H55B	108.3
C2—C1—N9	118.9 (3)	H55A—C55—H55B	107.4
C6—C1—N9	119.2 (3)	C55—C56—C57	110.7 (3)
C1—C2—C3	119.6 (3)	C55—C56—H56A	109.5
C1—C2—H2	120.2	C57—C56—H56A	109.5
C3—C2—H2	120.2	C55—C56—H56B	109.5
C2—C3—C4	120.0 (3)	C57—C56—H56B	109.5
C2—C3—H3	120.0	H56A—C56—H56B	108.1
C4—C3—H3	120.0	C56—C57—C58	112.4 (3)
C5—C4—C3	119.1 (3)	C56—C57—H57A	109.1
C5—C4—N8	117.2 (3)	C58—C57—H57A	109.1
C3—C4—N8	123.7 (3)	C56—C57—H57B	109.1
C4—C5—C6	121.1 (3)	C58—C57—H57B	109.1
C4—C5—H5	119.5	H57A—C57—H57B	107.9
C6—C5—H5	119.5	C57—C58—H58A	109.5
C1—C6—C5	118.4 (3)	C57—C58—H58B	109.5
C1—C6—H6	120.8	H58A—C58—H58B	109.5
C5—C6—H6	120.8	C57—C58—H58C	109.5
O4—C7—N4	121.4 (3)	H58A—C58—H58C	109.5
O4—C7—N8	122.6 (2)	H58B—C58—H58C	109.5
N4—C7—N8	116.0 (2)	C60—C59—N19	116.4 (2)
N4—C8—C9	112.0 (2)	C60—C59—H59A	108.2
N4—C8—H8A	109.2	N19—C59—H59A	108.2
C9—C8—H8A	109.2	C60—C59—H59B	108.2
N4—C8—H8B	109.2	N19—C59—H59B	108.2
C9—C8—H8B	109.2	H59A—C59—H59B	107.3
H8A—C8—H8B	107.9	C59—C60—C61	111.0 (3)
N3—C9—C8	111.5 (2)	C59—C60—H60A	109.4
N3—C9—H9A	109.3	C61—C60—H60A	109.4
C8—C9—H9A	109.3	C59—C60—H60B	109.4
N3—C9—H9B	109.3	C61—C60—H60B	109.4
C8—C9—H9B	109.3	H60A—C60—H60B	108.0
H9A—C9—H9B	108.0	C62—C61—C60	115.8 (3)
O3—C10—N3	123.7 (2)	C62—C61—H61A	108.3
O3—C10—N2	123.2 (2)	C60—C61—H61A	108.3
N3—C10—N2	113.1 (2)	C62—C61—H61B	108.3
N2—C11—C12	124.3 (2)	C60—C61—H61B	108.3
N2—C11—C16	117.3 (2)	H61A—C61—H61B	107.4
C12—C11—C16	118.4 (2)	C61—C62—H62A	109.5
C13—C12—C11	120.2 (3)	C61—C62—H62B	109.5
C13—C12—H12	119.9	H62A—C62—H62B	109.5
C11—C12—H12	119.9	C61—C62—H62C	109.5
C12—C13—C14	119.9 (3)	H62A—C62—H62C	109.5
C12—C13—H13	120.1	H62B—C62—H62C	109.5
C14—C13—H13	120.1	C64—C63—N19	116.6 (2)
C15—C14—C13	121.1 (3)	C64—C63—H63A	108.1
C15—C14—N1	119.3 (2)	N19—C63—H63A	108.1
C13—C14—N1	119.5 (3)	C64—C63—H63B	108.1
C16—C15—C14	119.2 (3)	N19—C63—H63B	108.1
C16—C15—H15	120.4	H63A—C63—H63B	107.3
C14—C15—H15	120.4	C65—C64—C63	111.0 (3)
C15—C16—C11	121.1 (3)	C65—C64—H64A	109.4
C15—C16—H16	119.4	C63—C64—H64A	109.4
C11—C16—H16	119.4	C65—C64—H64B	109.4
N4—C17—C18	112.3 (2)	C63—C64—H64B	109.4
N4—C17—H17A	109.2	H64A—C64—H64B	108.0
C18—C17—H17A	109.2	C64—C65—C66	114.6 (3)
N4—C17—H17B	109.2	C64—C65—H65A	108.6
C18—C17—H17B	109.2	C66—C65—H65A	108.6
H17A—C17—H17B	107.9	C64—C65—H65B	108.6
N5—C18—C17	111.0 (2)	C66—C65—H65B	108.6
N5—C18—H18A	109.4	H65A—C65—H65B	107.6
C17—C18—H18A	109.4	C65—C66—H66A	109.5
N5—C18—H18B	109.4	C65—C66—H66B	109.5
C17—C18—H18B	109.4	H66A—C66—H66B	109.5
H18A—C18—H18B	108.0	C65—C66—H66C	109.5
O7—C19—N5	122.3 (2)	H66A—C66—H66C	109.5
O7—C19—N6	124.2 (2)	H66B—C66—H66C	109.5
N5—C19—N6	113.6 (2)	C75—N20—C71	111.4 (2)
N6—C20—C21	123.7 (2)	C75—N20—C67	109.4 (2)
N6—C20—C25	117.3 (2)	C71—N20—C67	108.7 (2)
C21—C20—C25	119.0 (2)	C75—N20—C79	108.2 (2)
C22—C21—C20	119.5 (3)	C71—N20—C79	108.4 (3)
C22—C21—H21	120.3	C67—N20—C79	110.7 (2)
C20—C21—H21	120.3	C68—C67—N20	116.6 (3)
C23—C22—C21	120.3 (3)	C68—C67—H67A	108.1
C23—C22—H22	119.8	N20—C67—H67A	108.1
C21—C22—H22	119.8	C68—C67—H67B	108.1
C22—C23—C24	121.4 (3)	N20—C67—H67B	108.1
C22—C23—N7	119.7 (3)	H67A—C67—H67B	107.3
C24—C23—N7	118.9 (3)	C67—C68—C69	111.7 (3)
C25—C24—C23	118.6 (2)	C67—C68—H68A	109.3
C25—C24—H24	120.7	C69—C68—H68A	109.3
C23—C24—H24	120.7	C67—C68—H68B	109.3
C24—C25—C20	121.2 (2)	C69—C68—H68B	109.3
C24—C25—H25	119.4	H68A—C68—H68B	107.9
C20—C25—H25	119.4	C70—C69—C68	113.2 (3)
O10B—N10—O11B	121.8 (4)	C70—C69—H69A	108.9
O11A—N10—O10A	120.6 (5)	C68—C69—H69A	108.9
O11A—N10—C41	121.8 (5)	C70—C69—H69B	108.9
O10B—N10—C41	119.6 (3)	C68—C69—H69B	108.9
O11B—N10—C41	115.9 (4)	H69A—C69—H69B	107.8
O10A—N10—C41	115.8 (3)	C69—C70—H70A	109.5
C38—N11—C37	128.6 (2)	C69—C70—H70B	109.5
C38—N11—H11N	116 (2)	H70A—C70—H70B	109.5
C37—N11—H11N	115 (2)	C69—C70—H70C	109.5
C37—N12—C36	122.4 (2)	H70A—C70—H70C	109.5
C37—N12—H12N	122 (2)	H70B—C70—H70C	109.5
C36—N12—H12N	115 (2)	N20—C71—C72	115.0 (3)
C44—N13—C34	124.1 (2)	N20—C71—H71A	108.5
C44—N13—C35	117.9 (2)	C72—C71—H71A	108.5
C34—N13—C35	117.95 (19)	N20—C71—H71B	108.5
C32—N14—C33	119.4 (2)	C72—C71—H71B	108.5
C32—N14—H14N	120 (2)	H71A—C71—H71B	107.5
C33—N14—H14N	118 (2)	C73—C72—C71	112.4 (3)
C32—N15—C29	128.0 (2)	C73—C72—H72A	109.1
C32—N15—H15N	115 (2)	C71—C72—H72A	109.1
C29—N15—H15N	117 (2)	C73—C72—H72B	109.1
O18—N16—O17	123.3 (3)	C71—C72—H72B	109.1
O18—N16—C26	118.7 (3)	H72A—C72—H72B	107.9
O17—N16—C26	118.0 (3)	C72—C73—C74	112.1 (3)
C44—N17—C45	126.4 (2)	C72—C73—H73A	109.2
C44—N17—H17N	118.8 (19)	C74—C73—H73A	109.2
C45—N17—H17N	114.6 (19)	C72—C73—H73B	109.2
O14—N18—O15	123.2 (2)	C74—C73—H73B	109.2
O14—N18—C48	118.2 (2)	H73A—C73—H73B	107.9
O15—N18—C48	118.6 (2)	C73—C74—H74A	109.5
C27—C26—C31	121.1 (3)	C73—C74—H74B	109.5
C27—C26—N16	119.2 (3)	H74A—C74—H74B	109.5
C31—C26—N16	119.7 (3)	C73—C74—H74C	109.5
C26—C27—C28	118.8 (2)	H74A—C74—H74C	109.5
C26—C27—H27	120.6	H74B—C74—H74C	109.5
C28—C27—H27	120.6	C76—C75—N20	115.8 (3)
C27—C28—C29	121.3 (2)	C76—C75—H75A	108.3
C27—C28—H28	119.4	N20—C75—H75A	108.3
C29—C28—H28	119.4	C76—C75—H75B	108.3
C30—C29—N15	124.3 (2)	N20—C75—H75B	108.3
C30—C29—C28	118.9 (2)	H75A—C75—H75B	107.4
N15—C29—C28	116.8 (2)	C75—C76—C77	110.9 (3)
C31—C30—C29	119.7 (3)	C75—C76—H76A	109.5
C31—C30—H30	120.2	C77—C76—H76A	109.5
C29—C30—H30	120.2	C75—C76—H76B	109.5
C26—C31—C30	120.3 (3)	C77—C76—H76B	109.5
C26—C31—H31	119.8	H76A—C76—H76B	108.1
C30—C31—H31	119.8	C78—C77—C76	112.7 (3)
O16—C32—N14	122.2 (2)	C78—C77—H77A	109.1
O16—C32—N15	124.3 (2)	C76—C77—H77A	109.1
N14—C32—N15	113.6 (2)	C78—C77—H77B	109.1
N14—C33—C34	111.3 (2)	C76—C77—H77B	109.1
N14—C33—H33A	109.4	H77A—C77—H77B	107.8
C34—C33—H33A	109.4	C77—C78—H78A	109.5
N14—C33—H33B	109.4	C77—C78—H78B	109.5
C34—C33—H33B	109.4	H78A—C78—H78B	109.5
H33A—C33—H33B	108.0	C77—C78—H78C	109.5
N13—C34—C33	112.3 (2)	H78A—C78—H78C	109.5
N13—C34—H34A	109.2	H78B—C78—H78C	109.5
C33—C34—H34A	109.2	C80A—C79—N20	117.2 (3)
N13—C34—H34B	109.2	C80B—C79—N20	115.3 (4)
C33—C34—H34B	109.2	C80A—C79—H79A	108.0
H34A—C34—H34B	107.9	N20—C79—H79A	108.0
N13—C35—C36	112.4 (2)	C80A—C79—H79B	108.0
N13—C35—H35A	109.1	N20—C79—H79B	108.0
C36—C35—H35A	109.1	H79A—C79—H79B	107.2
N13—C35—H35B	109.1	C80B—C79—H79C	108.5
C36—C35—H35B	109.1	N20—C79—H79C	108.5
H35A—C35—H35B	107.9	C80B—C79—H79D	108.5
N12—C36—C35	111.2 (2)	N20—C79—H79D	108.5
N12—C36—H36A	109.4	H79C—C79—H79D	107.5
C35—C36—H36A	109.4	C81A—C80A—C79	111.0 (5)
N12—C36—H36B	109.4	C81A—C80A—H80A	109.4
C35—C36—H36B	109.4	C79—C80A—H80A	109.4
H36A—C36—H36B	108.0	C81A—C80A—H80B	109.4
O12—C37—N12	124.5 (2)	C79—C80A—H80B	109.4
O12—C37—N11	123.9 (2)	H80A—C80A—H80B	108.0
N12—C37—N11	111.6 (2)	C82A—C81A—C80A	119.5 (7)
N11—C38—C43	124.9 (2)	C82A—C81A—H81A	107.4
N11—C38—C39	116.5 (2)	C80A—C81A—H81A	107.4
C43—C38—C39	118.7 (2)	C82A—C81A—H81B	107.4
C40—C39—C38	121.2 (2)	C80A—C81A—H81B	107.4
C40—C39—H39	119.4	H81A—C81A—H81B	107.0
C38—C39—H39	119.4	C81A—C82A—H82A	109.5
C39—C40—C41	119.2 (3)	C81A—C82A—H82B	109.5
C39—C40—H40	120.4	H82A—C82A—H82B	109.5
C41—C40—H40	120.4	C81A—C82A—H82C	109.5
C40—C41—C42	121.1 (3)	H82A—C82A—H82C	109.5
C40—C41—N10	119.0 (3)	H82B—C82A—H82C	109.5
C42—C41—N10	119.9 (3)	C81B—C80B—C79	117.2 (5)
C43—C42—C41	120.3 (3)	C81B—C80B—H80C	108.0
C43—C42—H42	119.9	C79—C80B—H80C	108.0
C41—C42—H42	119.9	C81B—C80B—H80D	108.0
C42—C43—C38	119.5 (3)	C79—C80B—H80D	108.0
C42—C43—H43	120.3	H80C—C80B—H80D	107.2
C38—C43—H43	120.3	C82B—C81B—C80B	114.5 (7)
O13—C44—N13	121.2 (2)	C82B—C81B—H81C	108.6
O13—C44—N17	122.9 (2)	C80B—C81B—H81C	108.6
N13—C44—N17	115.9 (2)	C82B—C81B—H81D	108.6
C50—C45—C46	119.2 (2)	C80B—C81B—H81D	108.6
C50—C45—N17	116.4 (2)	H81C—C81B—H81D	107.6
C46—C45—N17	124.3 (2)	C81B—C82B—H82D	109.5
C47—C46—C45	119.8 (2)	C81B—C82B—H82E	109.5
C47—C46—H46	120.1	H82D—C82B—H82E	109.5
C45—C46—H46	120.1	C81B—C82B—H82F	109.5
C48—C47—C46	119.6 (2)	H82D—C82B—H82F	109.5
C48—C47—H47	120.2	H82E—C82B—H82F	109.5
C46—C47—H47	120.2	O22—P1—O19	114.54 (10)
C47—C48—C49	122.0 (2)	O22—P1—O20	111.33 (9)
C47—C48—N18	119.6 (2)	O19—P1—O20	107.35 (10)
C49—C48—N18	118.5 (2)	O22—P1—O21	106.44 (10)
C50—C49—C48	118.3 (2)	O19—P1—O21	109.89 (9)
C50—C49—H49	120.8	O20—P1—O21	107.06 (10)
C48—C49—H49	120.8	P1—O20—H20O	111 (2)
C49—C50—C45	121.0 (2)	P1—O21—H21O	115 (2)
C49—C50—H50	119.5	O24—P2—O26	112.39 (10)
C45—C50—H50	119.5	O24—P2—O25	108.25 (10)
C54—N19—C63	108.4 (2)	O26—P2—O25	110.73 (10)
C54—N19—C59	111.8 (2)	O24—P2—O23	110.98 (9)
C63—N19—C59	108.9 (2)	O26—P2—O23	106.28 (10)
C54—N19—C55	109.5 (2)	O25—P2—O23	108.14 (10)
C63—N19—C55	110.2 (2)	P2—O23—H23O	114 (2)
C59—N19—C55	108.1 (2)	P2—O25—H25O	113 (2)
C52—C51—H51A	109.5		
			
O6—N9—C1—C2	−166.3 (3)	C37—N12—C36—C35	−97.3 (3)
O5—N9—C1—C2	14.0 (4)	N13—C35—C36—N12	−172.7 (2)
O6—N9—C1—C6	12.2 (4)	C36—N12—C37—O12	−4.5 (4)
O5—N9—C1—C6	−167.5 (3)	C36—N12—C37—N11	175.5 (2)
C6—C1—C2—C3	−1.0 (5)	C38—N11—C37—O12	−4.1 (5)
N9—C1—C2—C3	177.5 (3)	C38—N11—C37—N12	175.9 (2)
C1—C2—C3—C4	1.4 (5)	C37—N11—C38—C43	−6.0 (4)
C2—C3—C4—C5	−0.9 (5)	C37—N11—C38—C39	174.8 (3)
C2—C3—C4—N8	177.3 (3)	N11—C38—C39—C40	179.6 (2)
C7—N8—C4—C5	−172.6 (3)	C43—C38—C39—C40	0.3 (4)
C7—N8—C4—C3	9.2 (5)	C38—C39—C40—C41	1.2 (4)
C3—C4—C5—C6	−0.1 (4)	C39—C40—C41—C42	−2.3 (4)
N8—C4—C5—C6	−178.3 (3)	C39—C40—C41—N10	178.7 (3)
C2—C1—C6—C5	0.0 (5)	O11A—N10—C41—C40	−2.6 (11)
N9—C1—C6—C5	−178.4 (3)	O10B—N10—C41—C40	−162.2 (4)
C4—C5—C6—C1	0.5 (4)	O11B—N10—C41—C40	−0.6 (9)
C17—N4—C7—O4	175.2 (3)	O10A—N10—C41—C40	161.9 (4)
C8—N4—C7—O4	−4.5 (4)	O11A—N10—C41—C42	178.3 (10)
C17—N4—C7—N8	−4.0 (4)	O10B—N10—C41—C42	18.8 (5)
C8—N4—C7—N8	176.4 (2)	O11B—N10—C41—C42	−179.6 (8)
C4—N8—C7—O4	4.9 (5)	O10A—N10—C41—C42	−17.2 (5)
C4—N8—C7—N4	−176.0 (3)	C40—C41—C42—C43	1.8 (5)
C7—N4—C8—C9	100.0 (3)	N10—C41—C42—C43	−179.2 (3)
C17—N4—C8—C9	−79.6 (3)	C41—C42—C43—C38	−0.2 (5)
C10—N3—C9—C8	91.6 (3)	N11—C38—C43—C42	180.0 (3)
N4—C8—C9—N3	177.7 (2)	C39—C38—C43—C42	−0.8 (4)
C9—N3—C10—O3	0.3 (4)	C34—N13—C44—O13	−179.1 (2)
C9—N3—C10—N2	−179.3 (2)	C35—N13—C44—O13	3.2 (4)
C11—N2—C10—O3	6.3 (4)	C34—N13—C44—N17	0.5 (4)
C11—N2—C10—N3	−174.0 (2)	C35—N13—C44—N17	−177.3 (2)
C10—N2—C11—C12	2.1 (4)	C45—N17—C44—O13	4.7 (4)
C10—N2—C11—C16	−179.0 (2)	C45—N17—C44—N13	−174.8 (2)
N2—C11—C12—C13	−179.0 (2)	C44—N17—C45—C50	−177.8 (2)
C16—C11—C12—C13	2.1 (4)	C44—N17—C45—C46	3.1 (4)
C11—C12—C13—C14	−0.3 (4)	C50—C45—C46—C47	0.9 (4)
C12—C13—C14—C15	−1.8 (4)	N17—C45—C46—C47	−179.9 (2)
C12—C13—C14—N1	175.4 (2)	C45—C46—C47—C48	−1.6 (4)
O2—N1—C14—C15	174.6 (3)	C46—C47—C48—C49	0.8 (4)
O1—N1—C14—C15	−2.7 (4)	C46—C47—C48—N18	−179.3 (2)
O2—N1—C14—C13	−2.6 (4)	O14—N18—C48—C47	−14.2 (4)
O1—N1—C14—C13	−179.9 (2)	O15—N18—C48—C47	165.5 (2)
C13—C14—C15—C16	2.0 (4)	O14—N18—C48—C49	165.7 (3)
N1—C14—C15—C16	−175.2 (2)	O15—N18—C48—C49	−14.6 (3)
C14—C15—C16—C11	−0.2 (4)	C47—C48—C49—C50	0.7 (4)
N2—C11—C16—C15	179.2 (2)	N18—C48—C49—C50	−179.3 (2)
C12—C11—C16—C15	−1.8 (4)	C48—C49—C50—C45	−1.3 (4)
C7—N4—C17—C18	81.5 (3)	C46—C45—C50—C49	0.6 (4)
C8—N4—C17—C18	−98.9 (3)	N17—C45—C50—C49	−178.7 (2)
C19—N5—C18—C17	75.3 (3)	C51—C52—C53—C54	−56.8 (4)
N4—C17—C18—N5	−161.0 (2)	C63—N19—C54—C53	−172.7 (2)
C18—N5—C19—O7	−5.2 (4)	C59—N19—C54—C53	−52.7 (3)
C18—N5—C19—N6	174.8 (2)	C55—N19—C54—C53	67.1 (3)
C20—N6—C19—O7	12.1 (4)	C52—C53—C54—N19	−167.5 (2)
C20—N6—C19—N5	−167.8 (3)	C54—N19—C55—C56	178.0 (2)
C19—N6—C20—C21	−22.3 (4)	C63—N19—C55—C56	58.9 (3)
C19—N6—C20—C25	157.0 (3)	C59—N19—C55—C56	−59.9 (3)
N6—C20—C21—C22	177.5 (3)	N19—C55—C56—C57	−173.0 (3)
C25—C20—C21—C22	−1.8 (4)	C55—C56—C57—C58	−174.2 (3)
C20—C21—C22—C23	1.2 (4)	C54—N19—C59—C60	−56.1 (3)
C21—C22—C23—C24	0.5 (4)	C63—N19—C59—C60	63.6 (3)
C21—C22—C23—N7	−178.2 (2)	C55—N19—C59—C60	−176.7 (3)
O8—N7—C23—C22	−172.3 (3)	N19—C59—C60—C61	−172.1 (3)
O9—N7—C23—C22	7.3 (4)	C59—C60—C61—C62	−179.5 (4)
O8—N7—C23—C24	8.9 (4)	C54—N19—C63—C64	−70.4 (3)
O9—N7—C23—C24	−171.4 (2)	C59—N19—C63—C64	167.8 (3)
C22—C23—C24—C25	−1.4 (4)	C55—N19—C63—C64	49.4 (3)
N7—C23—C24—C25	177.3 (2)	N19—C63—C64—C65	172.0 (3)
C23—C24—C25—C20	0.7 (4)	C63—C64—C65—C66	171.0 (3)
N6—C20—C25—C24	−178.4 (2)	C75—N20—C67—C68	52.6 (3)
C21—C20—C25—C24	0.9 (4)	C71—N20—C67—C68	174.5 (3)
O18—N16—C26—C27	−8.2 (4)	C79—N20—C67—C68	−66.5 (3)
O17—N16—C26—C27	171.0 (3)	N20—C67—C68—C69	173.3 (3)
O18—N16—C26—C31	173.4 (3)	C67—C68—C69—C70	171.4 (3)
O17—N16—C26—C31	−7.5 (5)	C75—N20—C71—C72	49.7 (3)
C31—C26—C27—C28	−0.4 (4)	C67—N20—C71—C72	−71.0 (3)
N16—C26—C27—C28	−178.8 (3)	C79—N20—C71—C72	168.6 (3)
C26—C27—C28—C29	−0.7 (4)	N20—C71—C72—C73	−153.1 (3)
C32—N15—C29—C30	16.0 (4)	C71—C72—C73—C74	175.4 (3)
C32—N15—C29—C28	−162.8 (2)	C71—N20—C75—C76	51.1 (3)
C27—C28—C29—C30	0.6 (4)	C67—N20—C75—C76	171.3 (2)
C27—C28—C29—N15	179.6 (2)	C79—N20—C75—C76	−68.1 (3)
N15—C29—C30—C31	−178.3 (3)	N20—C75—C76—C77	−166.4 (3)
C28—C29—C30—C31	0.5 (5)	C75—C76—C77—C78	74.3 (4)
C27—C26—C31—C30	1.5 (5)	C75—N20—C79—C80A	−163.3 (6)
N16—C26—C31—C30	179.9 (3)	C71—N20—C79—C80A	75.7 (6)
C29—C30—C31—C26	−1.5 (5)	C67—N20—C79—C80A	−43.4 (6)
C33—N14—C32—O16	3.9 (4)	C75—N20—C79—C80B	172.4 (5)
C33—N14—C32—N15	−175.8 (2)	C71—N20—C79—C80B	51.4 (5)
C29—N15—C32—O16	−6.2 (4)	C67—N20—C79—C80B	−67.8 (5)
C29—N15—C32—N14	173.5 (2)	C80B—C79—C80A—C81A	−84.0 (13)
C32—N14—C33—C34	−75.5 (3)	N20—C79—C80A—C81A	−174.5 (5)
C44—N13—C34—C33	−81.5 (3)	C79—C80A—C81A—C82A	53.5 (12)
C35—N13—C34—C33	96.2 (3)	C80A—C79—C80B—C81B	108.0 (17)
N14—C33—C34—N13	164.1 (2)	N20—C79—C80B—C81B	−151.7 (6)
C44—N13—C35—C36	−87.5 (3)	C79—C80B—C81B—C82B	−46.3 (11)
C34—N13—C35—C36	94.6 (3)		

### Hydrogen-bond geometry (Å, º)

**Table:**

*D*—H···*A*	*D*—H	H···*A*	*D*···*A*	*D*—H···*A*
N8—H8*N*···O7	0.90 (3)	2.07 (3)	2.955 (3)	168 (3)
N17—H17*N*···O16	0.90 (2)	2.21 (2)	3.100 (3)	170 (2)
N5—H5*N*···O21	0.90 (2)	2.04 (2)	2.915 (3)	164 (3)
N6—H6*N*···O22	0.90 (2)	2.10 (2)	2.986 (3)	168 (2)
N11—H11*N*···O19	0.90 (2)	1.96 (2)	2.854 (3)	175 (2)
N12—H12*N*···O22	0.90 (2)	2.21 (2)	3.058 (3)	157 (3)
O20—H20*O*···O24	0.93 (2)	1.66 (2)	2.583 (2)	178 (3)
O23—H23*O*···O22	0.93 (2)	1.64 (2)	2.568 (2)	176 (2)
O21—H21*O*···O19^i^	0.93 (2)	1.61 (2)	2.528 (2)	173 (3)
O25—H25*O*···O26^ii^	0.93 (2)	1.59 (2)	2.501 (2)	166 (3)
N2—H2*N*···O26^i^	0.90 (2)	2.08 (2)	2.977 (3)	174 (2)
N3—H3*N*···O24^i^	0.90 (2)	2.09 (2)	2.931 (3)	155 (3)
N14—H14*N*···O25^ii^	0.90 (2)	2.03 (2)	2.930 (3)	175 (2)
N15—H15*N*···O24^ii^	0.90 (2)	2.10 (2)	2.986 (3)	168 (2)
C3—H3···O4	0.95	2.17	2.789 (4)	121
C5—H5···O7	0.95	2.53	3.279 (4)	136
C12—H12···O3	0.95	2.23	2.844 (3)	122
C21—H21···O7	0.95	2.31	2.872 (4)	118
C30—H30···O16	0.95	2.32	2.903 (4)	119
C43—H43···O12	0.95	2.32	2.910 (4)	120
C46—H46···O13	0.95	2.19	2.810 (3)	122
C18—H18*A*···O1^iii^	0.99	2.42	3.345 (4)	156
C30—H30···O6^iv^	0.95	2.58	3.265 (4)	129
C33—H33*B*···O11*A*^iii^	0.99	2.58	3.235 (12)	123
C39—H39···O21^i^	0.95	2.52	3.446 (3)	164
C54—H54*A*···O15^v^	0.99	2.46	3.447 (4)	173
C63—H63*A*···O3	0.99	2.42	3.379 (4)	164
C63—H63*B*···O4	0.99	2.26	3.003 (4)	131
C64—H64*B*···O15^v^	0.99	2.46	3.348 (4)	149
C70—H70*B*···O10*A*^iii^	0.98	2.47	3.289 (6)	141
C75—H75*A*···O13	0.99	2.31	3.158 (4)	143
C75—H75*B*···O1^vi^	0.99	2.58	3.514 (4)	157

Symmetry codes: (i) −*x*+1, −*y*+2, −*z*+1; (ii) −*x*+2, −*y*+2, −*z*+1; (iii) *x*+1, *y*, *z*; (iv) −*x*+2, −*y*+1, −*z*+1; (v) *x*−1, *y*, *z*−1; (vi) *x*+1, *y*, *z*+1.
